# Multifaceted role of mTOR (mammalian target of rapamycin) signaling pathway in human health and disease

**DOI:** 10.1038/s41392-023-01608-z

**Published:** 2023-10-02

**Authors:** Vivek Panwar, Aishwarya Singh, Manini Bhatt, Rajiv K. Tonk, Shavkatjon Azizov, Agha Saquib Raza, Shinjinee Sengupta, Deepak Kumar, Manoj Garg

**Affiliations:** 1 Department of Pharmaceutical Chemistry, School of Pharmaceutical Sciences, Shoolini University, Solan, Himachal Pradesh 173229 India; 2 Amity Institute of Molecular Medicine and Stem Cell Research (AIMMSCR), Amity University Uttar Pradesh, Sector-125, Noida, Uttar Pradesh 201313 India; 3 Department of Biomedical Engineering, Indian Institute of Technology, Ropar, Punjab 140001 India; 4 Department of Pharmaceutical Chemistry, School of Pharmaceutical Sciences, Delhi Pharmaceutical Sciences and Research University (DPSRU), New Delhi, 110017 India; 5 Laboratory of Biological Active Macromolecular Systems, Institute of Bioorganic Chemistry, Academy of Sciences Uzbekistan, Tashkent, 100125 Uzbekistan; 6 Faculty of Life Sciences, Pharmaceutical Technical University, 100084 Tashkent, Uzbekistan; 7 Rajive Gandhi Super Speciality Hospital, Tahirpur, New Delhi 110093 India

**Keywords:** Cancer, Cell biology

## Abstract

The mammalian target of rapamycin (mTOR) is a protein kinase that controls cellular metabolism, catabolism, immune responses, autophagy, survival, proliferation, and migration, to maintain cellular homeostasis. The mTOR signaling cascade consists of two distinct multi-subunit complexes named mTOR complex 1/2 (mTORC1/2). mTOR catalyzes the phosphorylation of several critical proteins like AKT, protein kinase C, insulin growth factor receptor (IGF-1R), 4E binding protein 1 (4E-BP1), ribosomal protein S6 kinase (S6K), transcription factor EB (TFEB), sterol-responsive element-binding proteins (SREBPs), Lipin-1, and Unc-51-like autophagy-activating kinases. mTOR signaling plays a central role in regulating translation, lipid synthesis, nucleotide synthesis, biogenesis of lysosomes, nutrient sensing, and growth factor signaling. The emerging pieces of evidence have revealed that the constitutive activation of the mTOR pathway due to mutations/amplification/deletion in either mTOR and its complexes (mTORC1 and mTORC2) or upstream targets is responsible for aging, neurological diseases, and human malignancies. Here, we provide the detailed structure of mTOR, its complexes, and the comprehensive role of upstream regulators, as well as downstream effectors of mTOR signaling cascades in the metabolism, biogenesis of biomolecules, immune responses, and autophagy. Additionally, we summarize the potential of long noncoding RNAs (lncRNAs) as an important modulator of mTOR signaling. Importantly, we have highlighted the potential of mTOR signaling in aging, neurological disorders, human cancers, cancer stem cells, and drug resistance. Here, we discuss the developments for the therapeutic targeting of mTOR signaling with improved anticancer efficacy for the benefit of cancer patients in clinics.

## Introduction

The mTOR belongs to the class of evolutionarily conserved threonine and serine kinases which recognize and incorporate a variety of extracellular and intracellular signals to maintain cellular homeostasis and metabolism.^1–4^ The name mTOR was obtained from rapamycin isolated from a soil bacterium in 1970 on Rapa Nui.^1–4^ Further, the structural elucidation of the rapamycin revealed 14–16 membered lactone rings and reduced saccharide substituents. Interestingly, the physiological characterizations have uncovered immunosuppressive properties, curtailed organ rejection, kidney transplantation, and inhibition of T-cell mitogenesis.^2,5^ Mechanistically, the mTOR has dual kinase activity and can phosphorylate serine/threonine or tyrosine residue. The mTOR has been considered a part of the phosphoinositide 3-kinase (PI3K) family due to the presence of the catalytic domain within the mTOR structure which has a similarity with lipid kinases like PI3K. mTOR has been reported to be crucial for many biological processes, including cell growth, cell survival, immunity, autophagy, and metabolism.^1,2^

This has been reported that mTOR can generate two different functional complexes named mTORC1 and mTORC2.^6^ The mTORC1 was discovered as a complex of several proteins that consist of mTOR, Raptor (regulatory associated protein of mTOR), GβL (G protein β subunit-like protein)/mLST8 (mammalian lethal with SEC13 protein 8), DEPTOR (DEP-domain-containing mTOR-interacting protein), and PRAS40 (the 40 kDa proline-rich Akt substrate).^7,8^ The mTORC2 is composed of mTOR, GβL/mLST8, Rictor (rapamycin-insensitive companion of mTOR), Protor/PRR5 (Proline-rich protein 5), DEPTOR, and mSIN1 (mammalian stress-activated protein kinase-interacting protein 1).^7–14^ The mTORC1 amalgamate signals from a variety of growth factors, and nutrients to enhance cellular proliferation especially when there is adequate energy and/or catabolism when the body is hungry.^5,15^ The mTORC1 is well known for its function in cell growth and metabolism, whereas the mTORC2 regulates proliferation and survival.^1^ Several groups reported that mTOR is crucial for several signaling cascades like AKT, PI3K, TSC1/TSC2 (tuberous sclerosis complex subunit 1 and 2), Rheb, LKBL/AMP-activated protein kinase (AMPK), VAM6/Rag GTPases.^16^ The mTOR signaling was demonstrated to enhance gene transcription and translation to control cellular growth, autophagy, and apoptosis.^2,5,17^

**Fig. 1:**
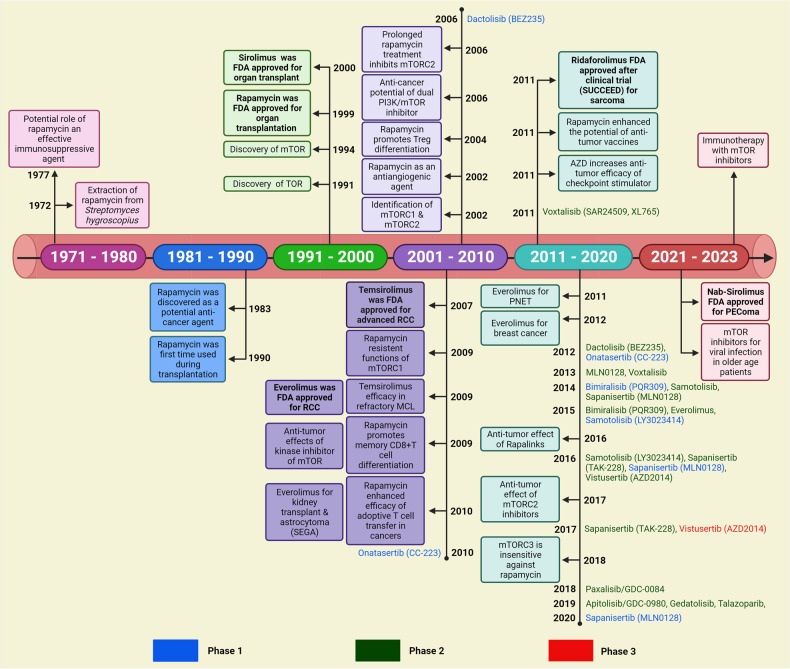
History of research on the discovery and development of mTOR signaling. The figure describes the journey of mTOR signaling from its origin to the most advanced scientific discoveries including the identification, isolation, development of inhibitors, and their application as therapeutics in human health and diseases. Created with BioRender.com. FDA Food and Drug Administration, TOR target of rapamycin, mTOR mammalian target of rapamycin, mTORC1 mTOR complex 1, mTORC2 mTOR complex 2, MCL mental cell lymphoma, Nab-sirolimus nanoparticle albumin-bound sirolimus, PEComa perivascular epithelioid cell tumor, PNET pancreatic neuroendocrine tumor, RCC renal cell carcinoma, SEGA subependymal giant-cell astrocytoma

Dysregulation of mTOR has been found to be strongly linked with several diseases like aging, arthritis, insulin resistance, osteoporosis, cancers, and neurological disorders.^18^ Cancer development is a complex, and multifactorial process, including genetic aberration, epigenetic modifications, dysregulated expression of hormones, tumor suppressors, and conversion of proto-oncogenes to oncogenes.^19–21^ The frequent alteration of mTOR was noticed to play an important role during tumorigenesis, distant metastasis, and drug resistance in human malignancies, such as lung, breast, liver, renal, pancreatic, and prostate.^22–25^ The stimulation of the mTOR cascade has been displayed to increase tumor growth through the regulation of glycolysis, angiogenesis, growth factor receptor pathway, lipid metabolism, and autophagy.^5,9^ Therefore, mTOR represents an important and promising target for therapeutic intervention against human malignancies.^26,27^ In this current review, we have discussed the structure of mTOR complexes along with their molecular functions, upstream regulators, as well as downstream effectors of mTOR signaling, the association of mTOR signaling to modulate cellular metabolism and autophagy. Also, how the dysregulated mTOR signaling is associated with aging, neurological disorders, and cancers (Fig. 1). Importantly, we have highlighted the opportunities and challenges for pharmacological targeting of mTOR signaling for therapeutic intervention and management of human malignancies.

## Structural architects of mTOR and its complexes

Mechanobiology of mTOR unfolds it as a complex protein kinase intricated with multielement complexes via its communication network with other proteins. The cryo-electron microscopic structure of the mTOR has revealed a hollow rhomboid architecture with dimensions of ~280 × 210 × 130 (Å^3^).^28^ This has been noticed that the C-terminus of mTOR was comprised of the FAT domain (FKBP12 rapamycin-associated protein, ataxia telangiectasia, and transformation or transactivation domain associated protein), FRB (FKBP12 rapamycin binding) domain which is responsible for the interaction of FKBP protein bound to rapamycin with mTOR, kinase domain that is an important site for phosphorylation to control the activity of mTOR, and FATC (FAT carboxyterminal domain). Also, the N-terminal part of the mTOR consists of 20 HEAT repeats. The HEAT repeats were found to be essential for interaction with Raptor and Rictor.^22,29^

The structural architect of mTORC1 defines the complex and symmetric organization of Raptor, PRAS40, DEPTOR, and mLST8 (GβL) as its major components along with centrally located mTOR protein.^30^ Interlocking interactions allying the two mTOR and two Raptor subunits configure dimeric interfaces. Distal foot-like perturbances of mLST8 (GβL) subunit interpose mTOR inside complex 1. PRAS40 circumscribes itself to the adjoining adjacency of Raptor subunits i.e., the middle section of the central core of complex 1.^31,32^ Raptor is critical for the assembly, proper localization, and stability of the mTORC1. Raptor was reported to be important for the recruitment of the substrate on mTORC1. PRAS40 has been shown to inhibit the activation of mTORC1 unless it is phosphorylated through growth factor receptor signaling by growth factors/other stimuli. PRAS40 has an essential role in human cancers and metabolic disorders. The mLST8 was found to be associated with the kinase domain of the mTORC1 and can help in the stabilization of kinase activity. DEPTOR acts as an inhibitory subunit in the mTORC1. The crystal structure and functional analysis revealed that rapamycin-FKBP12 can efficiently bind with the FRB domain of mTOR to obstruct the substrates from active sites.^1,29,31–33^ The mTORC1 has been found to control cellular growth by increasing the biogenesis of ribosomes, mRNA translation, and autophagy.^34^

The mTORC2 complex is a hollow rhombohedral fold with dimensions of 220 Å × 200 Å × 130Å.^28^ The complex embraces binary symmetry, and each promoter incorporates one copy of mTOR, GβL/mLST8, mSin1, and Rictor. mTOR-mLST8 (GβL) heterodimer embraces overall architecture alike to complex 1 with a root mean square deviation of 6.7 Å for 3550 α-carbon atoms.^30,35^ The two monomers of mTOR pack against each other to form a central scaffold yielding a binding surface for the other three components. Two copies of mLST8 (GβL), mSin1, or Rictor bind symmetrically to the mTOR dimer.^31,32^ Rictor is important for the assembly, substrate recognition, and stability of the mTORC2. This has been observed that mSIN1 acts as a scaffold protein that helps in the mTORC2 interaction with serum and glucocorticoid-activated kinase 1 (SGK1) and negatively controls the kinase activity of mTORC2.^12^ Protor-1, a Rictor-binding protein was found to regulate mTORC2-dependent phosphorylation of SGK1.^36^ The mTORC2 has been displayed to be associated with the cytoskeleton, cell proliferation, cell survival, and migration.

**Fig. 2:**
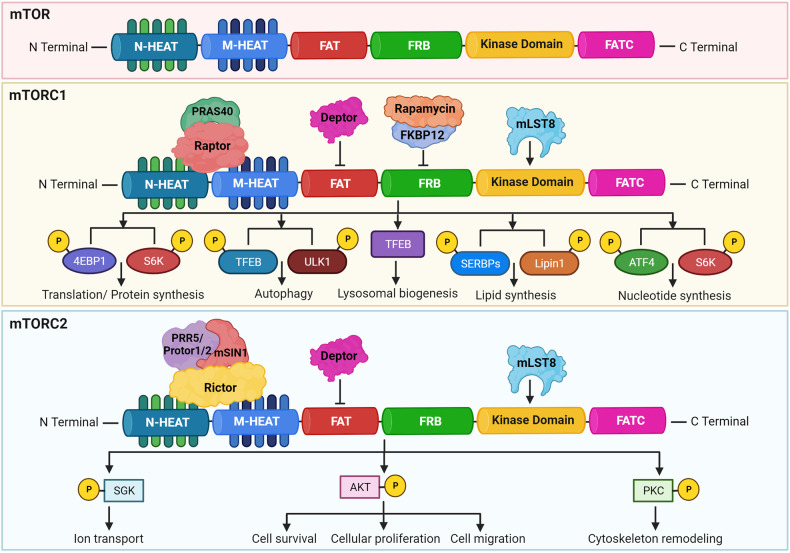
The domain structures of mTORC1 and mTORC2, their downstream signaling targets and functional role. N-terminal domain of mTOR possesses tandem HEAT repeats and C-terminal domains composed of FATC, kinase, FRB, and FAT. The mTOR signaling pathway is majorly constituted of two distinctive mTOR complexes named mTORC1 and mTORC2. The mTORC1 is a complex of DEPTOR, Raptor, PRAS40, mLST8, mTOR, and phosphorylate downstream targets to regulate protein synthesis or mRNA translation, lipid synthesis, nucleotide synthesis, lysosomal biogenesis, and autophagy. The mTORC2 is a complex of mTOR, DEPTOR, mSIN1, Rictor, Protor, and mLST8 to regulate cell survival, proliferation, migration, and cytoskeleton remodeling. Created with BioRender.com

Harwood et al. have identified another rapamycin-insensitive complex known as mTORC3. The E26 transformation-specific transcription factor ETV7 was found to interact with mTOR in the cytoplasmic compartment. The mTORC3 displayed bimodal mTORC1/2 activity that was independent of the components of mTORC1/2. This was noticed that mTORC3 is robustly activated in several cancers. The loss of mTORC3 expression in cancer cells displayed marked sensitivity to rapamycin. Interestingly, this study also demonstrated that mTORC3 induced tumorigenesis in a murine model of rhabdomyosarcoma. Interestingly, the transgenic ETV7 expression further enhanced tumor onset and penetrance.^37^ The detailed structure of the mTOR and its complexes, along with their function, has been described in Fig. 2.

## Upstream regulators of mTOR signaling

**Fig. 3:**
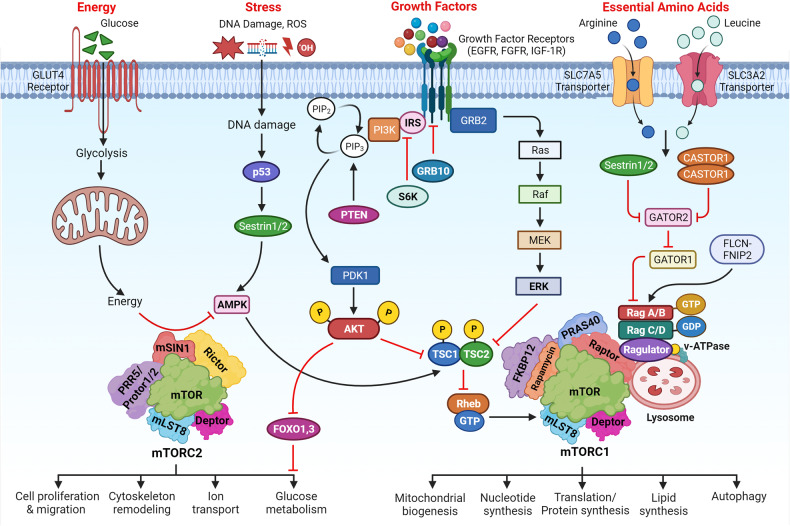
The major upstream regulators of mTORC1 and mTORC2. Growth factors, amino acids like arginine and leucine, energy from glucose or other sources, cell stresses including DNA damage, and ROS stimulate mTORC1 to modulate various biological processes like mitochondrial biogenesis, nucleotide synthesis, mRNA translation (protein synthesis), lipid synthesis, and autophagy. The growth factors are the main regulators of the mTORC2 to control cell proliferation, migration, cytoskeleton remodeling, ion transport, and glucose metabolism. Created with BioRender.com

The mTOR signaling cascades control the cellular growth and mitotic divisions by generating prominent metabolic energy from glucose, lipid, protein, and nucleotides while inhibiting catabolic processes like autophagy.^38^ Hence, the mTORC1 plays a central role in maintaining the equilibrium between anabolism and catabolism, especially in response to environmental stress. mTOR signaling augments energy depots and consumption. mTORC1 can enhance cellular growth by integrating stimuli from growth factors, DNA damage, oxygen, nutrients, amino acids, and energy, whereas augmented mTORC1 prompts insulin resistance by halting insulin receptor signaling and fat accumulation (Fig. 3).^1,38^ This has been observed that dysregulated oncogenes, enhanced anabolism, angiogenesis, and suppression of autophagy underlie the tumorigenic behavior of mTORC1. mTORC2 is activated by growth factors which in turn activates AKT and AGC family leading to increased cellular proliferation and survival.^1,38^ Growth factors are known to regulate several signaling cascades that intersect on TSC, including receptor tyrosine kinases (RTK), IGF-1, Wnt, TNFα, inflammatory cytokines, and Ras signaling cascade. These growth factors stimulate the phosphorylation of TSC2 through AKT. The phosphorylation was associated with the inhibition of TSC through its dissociation from the lysosomal membrane. The RTK-mediated Ras signaling was found to activate the mTORC1 pathway through MAPK/ERK and its effector p90^RSK^ leading phosphorylation of TSC2. Moreover, the other growth factors like Wnt, and TNFα were noticed with the activation of mTORC1 through repression of TSC1.^39^ Importantly, the environmental/extracellular and intracellular stresses are sensed by the mTORC1 and modulate cellular growth and survival under hypoxia, lower levels of ATP, and DNA damage conditions.^40^ Under glucose deprivation conditions, there is a marked reduction in cellular energy levels that stimulates energy stress- sensing metabolic regulator AMPK. The stimulation of AMPK was reported to repress the mTORC1 either through direct phosphorylation of Raptor or indirectly by phosphorylating TSC2.^41^ Furthermore, low glucose levels were found to suppress mTORC1 signaling by inhibiting the Rag GTPases activity, especially in cells lacking AMPK. Recently, Dai et al. have reported that AMPK-dependent phosphorylation of WDR24 can modulate the glucose-dependent activation of mTORC1.^42^ These results indicated that mTORC1 can efficiently sense glucose or energy stress through multiple molecular mechanisms.^43,44^ Hypoxia or oxygen deprivation stress was reported to produce an inhibitory effect on the mTORC1 through activation of AMPK, and induction of REDD1 that resulted in the activation of TSC. Also, the induction of the DNA damage response signaling was found to repress mTORC1 via induction of p53 target genes like *AMPKβ*, *PTEN*, and *TSC2* leading to increased activation of TSC activity. Amino acids are not only required as the building blocks of proteins but also a great source of carbon and energy for a variety of metabolic signaling cascades.^45^ The activation of the mTORC1 pathway is coupled with diet-mediated changes in the concentration of amino acids. Interestingly, the mechanism of sensing the amino acids through mTORC1 with the help of Rag GTPases as essential components of mTORC1 signaling was one of the groundbreaking discoveries in the field of mTOR signaling.^46^ Rags were discovered as heterodimers of RagA/RagB with RagC/RagD. These are mostly bounded by the membrane of the lysosome through their close association or interaction with a well-characterized pentameric complex composed of p14, HBXIP, p18, MP1, and c7ORF59.^47,48^ The amino acid stimulation converts the Rags to an active nucleotide-bound state which allows Rags to bind with Raptor and recruit mTORC1 on the surface of the lysosome, where Rheb is localized. The mTORC1 has been found to sense cytosolic as well as intra-lysosomal amino acids through different molecular mechanisms.^49,50^ SLC38A9, lysosomal amino acid transporter was displayed to interact with the Rag-v-ATPase complex which is responsible for arginine transport and activation of mTORC1.^50,51^ The cytosolic arginine and leucine signal to mTORC1 through GATOR1 and GATOR2 complexes. The GATOR1 is comprised of Nprl2/3, DEPDC5 and acts as a GAP for RagA/B to inhibit the mTORC1 pathway. The KICSTOR complex comprised of Kaptin, c12orf66, ITFG2, and SZT2 and bound to GATOR1 on the surface of the lysosome to modulate the mTORC1 pathway for nutrient/amino acids sensing.^52^ On the other hand, GATOR2 was discovered as a pentameric complex of WDR24, Seh1L, Mios, WDR59, and Sec13. GATOR2 interacts with GATOR1 on the surface of the lysosome as a positive regulator of the mTORC1 pathway.^53^ Sestrin2 was discovered as a GATOR2 interacting protein partner that senses cytosolic amino acid. This led to the inhibition of mTORC1 signaling under the deprivation of the amino acid.^54^ Structural and biochemical analyses revealed that Sestrin2 is a direct sensor of leucine and upstream of mTORC1. Further, Sestrin2 was shown to be transcriptionally induced after prolonged amino acid starvation through ATF-4. These results indicated that Sestrin2 can function as an acute leucine sensor and indirect mediator of prolonged starvation of amino acid.^55^ Arginine activated mTORC1 via the GATOR1/2-Rag pathway and binding the CASTOR1. CASTOR1 interacts and suppress GATOR2 in the absence of arginine and dissociates upon arginine binding resulting in the mTORC1 activation.^56,57^ These findings verified that CASTOR1 has the arginine sensing ability for the mTORC1 pathway. Additionally, other molecular mechanisms that control amino acids mediated mTORC1 signaling were reported and recruitment of Folliculin-FNIP2 complex on the lysosome cooperates as a GAP for RagC/D in the presence of amino acids.^58^ The glutamine is utilized as a source of nitrogen by highly dividing cells to stimulate mTORC1 independent of the Rag GTPases via Arf family GTPases.^59^ The long noncoding RNA-SPAR was found to cooperate with the v-ATPase-Ragulator complex. This cooperation caused a hamper in the process of the mTORC1 recruitment to lysosomes.^60^ Recently, Yan and colleagues have performed genome-wide CRISPR-Cas9 screening and identified interleukin enhancer binding factor 3 (ILF3) as a critical regulator for the sensing of the amino acids in a mTORC1-dependent manner. ILF3 was found to tether with GATOR complexes on the lysosomes. Further, the addition of the sequences that specifically target lysosome to the GATOR2 component WDR24 was found to bypass the ILF3 requirement and modulated the amino acid-mediated mTORC1 pathway.^61^ Another study by Jiang and colleagues demonstrated that WDR24 or Ring domains were critical for GATOR2 to disseminate amino acids availability to mTORC1 during embryonic development.^62^ Further, studies have confirmed the potential of mTORC2 as an effector of IGF and PI3K signaling. The mSin1 of the mTORC2 was found to have a phosphoinositide-binding domain which proved to be important for the insulin-regulated activity of the mTORC2. In the insufficiency of insulin, the PH domain of the mSin1 was noticed to hamper the mTORC2 catalytic activity. This autoinhibition was rescued upon binding to PI3K-generated PIP_3_^63^ and mSin1 was phosphorylated by AKT, indicating the existence of a positive-feedback loop that partially activates Akt and promoted the activation of mTORC2.^64^ The S6K1 was found to inhibit the mTORC2 signaling through the degradation of IRS1, insulin receptor substrate-1.^65^ The negative feedback loop among insulin-dependent PI3K pathway and mTORC1 is another mechanism for the mTOR2 regulation (Fig. 3). The mTORC1 was found to phosphorylate Grb10 (negative regulator of IGF-1R signaling) that is upstream to AKT and mTORC2.^66,67^ During the past several decades, the emerging pieces of evidence have provided valuable information about the regulation of the mTOR signaling that leads to the development of several clinical drugs. However, the complete knowledge of the integration of a variety of signals through TSC to regulate mTORC1/2 activity remains an open research question in the field.

### Role of the mTOR signaling cascade in glucose metabolism

The energy requirement of the cell is regulated by mTORC1 by AMPK, the sensor of intracellular energy levels. Augmented glucose metabolism promotes mitochondrial activity by prompting AMP levels that disturb the ATP: AMP ratio resulting in the activation of the AMPK leading to phosphorylation of TSC2 which enhances the GAP activity of TSC2 for the Rheb to repress the mTORC1 activity.^68^ Moreover, AMPK can directly phosphorylate Raptor and reduce mTORC1 activity under energy-deprived conditions.^41^ Also, mTORC1 can favor cellular proliferation through a prominent shift in glucose metabolism from oxidative phosphorylation to glycolysis. This metabolic reprogramming is known as the Warburg effect and is characterized by an increase in the uptake of glucose and the production of lactate, even in the presence of oxygen (aerobic glycolysis).^69^ mTORC1 enhanced the Warburg effect by increasing the gene expression and catalytic activity of key enzymes required during glycolysis, such as pyruvate kinase muscle isozyme 2, hexokinase 2, and lactate dehydrogenase A, among others. This results in increased flux through the glycolytic pathway, which provides the building blocks and energy required for cell growth and division.^69–71^ Hypoxic stress or anaerobic condition has been found to promote the reduction of pyruvate to lactate by NADH through lactate dehydrogenase that may augment the lactic acid concentration leading to lactic acidosis. Lactic acidosis favors oncogenesis by modulating the tumor microenvironment. Hypoxia-inducible factor 1 alpha (HIF-1α) was reported as a well-known regulator to increase the expression of the glycolytic enzymes, and glucose transporters.^70,71^ Glucose transporters thus facilitate the transport of glucose into cells while glycolytic enzymes catalyze the breakdown of glucose into energy.^69–72^ Moreover, mTORC1 has been found to enhance the translation of the HIF-1α which in turn activates the expression of key enzymes involved in glycolysis like phospho-fructokinase. The mTORC1-mediated SREBP stimulation caused increased flux by the pentose phosphate pathway leading to the generation of NADPH from the glucose and other intermediate metabolites for proliferation and growth.^56^

### Role of the mTOR signaling cascade in lipid metabolism

mTOR plays a significant role in regulating lipid biosynthesis required for cell growth and division by maintaining the cellular membrane. The mTORC1 has been shown to enhance the synthesis of lipids through the regulation of SREBP (sterol regulatory element-binding protein) that modulates the expression of the genes associated with cholesterol and fatty acid biosynthesis.^56^ Generally, during lower levels of sterol, the SREBP gets activated. The mTORC1 signaling cascade was reported to stimulate in two ways (1) SREBP activation via the S6K1 mechanism, and (2) through phosphorylation of the Lipin-1 that can repress SREBP activity in the absence of mTORC1.^56,73^ In the first mechanism, mTORC1 activates SREBP through the downstream effector S6K1, which phosphorylates and activates SREBP cleavage-activating protein (SCAP). This leads to the translocation of the active form of SREBP to the nucleus and the upregulation of genes involved in lipid synthesis. In the second mechanism, mTORC1 phosphorylates and inactivates Lipin-1, which is a negative regulator of SREBP activity.^73^ In the absence of mTORC1 signaling, Lipin-1 represses SREBP activity by promoting the formation of a repressor complex that inhibits SREBP-mediated transcription. However, under conditions of mTORC1 activation, Lipin-1 is phosphorylated and inactivated, leading to the derepression of SREBP activity and the upregulation of lipid biosynthesis.^73^ Overall, the regulation of SREBP by mTORC1 provides an important mechanism by which mTORC1 can promote lipid synthesis and support cellular growth and proliferation (Figs. 2 and 3).

### Role of the mTOR signaling cascade in nucleotide biogenesis

This has been confirmed that mTORC1 can promote the biosynthesis of nucleotides, especially in the proliferative cells to support the replication of the DNA and biogenesis of ribosomes. Moreover, mTORC1 was displayed to enhance the expression of MTHFD2 (methylenetetrahydrofolate dehydrogenase 2) in an ATF-4-dependent manner that can provide carbon units for the synthesis of purine. Ben-Sahra and colleagues have shown that S6K1 phosphorylates and stimulates carbamoyl phosphate synthetase (CAD) that helps in the pyrimidine synthesis pathway.^74^ Robitaille and colleagues have performed quantitative phosphoproteomics and identified that mTOR regulates the phosphorylation of approximately 335 proteins.^75^ This study showed that mTORC1 can phosphorylate CAD at serine 1859 via S6K and activated de novo synthesis of pyrimidines leading to the cell cycle through the S phase (Figs. 2 and 3). Therefore, mTORC1 regulates the production of nucleotides to adjust the RNA and DNA synthesis required for ribosome biogenesis.^75^

### Role of mTOR signaling in protein biogenesis

mTOR signaling cascade is well known for protein synthesis through the phosphorylation of eIF4E binding protein (4EBP), and S6K1. mTORC1 can phosphorylate S6K1 at Thr389 residue which leads to its phosphorylation and activation via PDK1 (3-phosphoinositide-dependent protein kinase 1). S6K1 can lead to the phosphorylation and activation of a variety of substrates that promote mRNA translation initiation, particularly the eIF4B which is crucial for the 5′cap binding eIF4F complex.^76^ Dorrello and colleagues have revealed that programmed cell death protein 4 (PDCD4) repressed the translation initiation factor eIF4A. During mitogen response, the phosphorylation of PDCD4 at Ser67 by the S6K1 leading to its degradation through the ubiquitin ligase SCF (β-TRCP) enables the synthesis of protein synthesis and cellular proliferation.^77^ Exon junction complex has been displayed to regulate mRNA synthesis.^78^ The SKAR-dependent recruitment of S6K1 to the newly generated mRNPs acts as a bridge between mTOR signaling and translation. The mTOR kinase has been recognized as one of the master regulators of translation to meet the demand for cancer cells. The global ribosome profiling was performed to unravel the mechanism of translation that regulates gene expression through mTOR in cancers. This study showed the enrichment of the specific genes associated with cellular growth, invasion, and metabolism. These genes were downstream targets of mTOR signaling in prostate cancer.^79^ Moreover, the potent and ATP-competitive mTOR inhibitor repressed mRNA translation and suppressed cellular proliferation.^80^ Another study has revealed that mRNAs that are controlled by mTORC1 are 5’ terminal oligopyrimidine (TOP) motifs. Moreover, the 4EBPs suppressed the initiation of translation by hampering the interaction between eIF4E and eIF4G1. This diminished the ability of eIF4E to interact with TOP and TOP-like mRNAs which explains why mTOR inhibition selectively repressed their translation.^81^ The mTORC1 phosphorylates its substrate 4EBP that triggers its dissociation from eIF4E allowing 5′cap-dependent mRNA translation (Figs. 2 and 3).^82,83^

## mTOR signaling in human cancers

The deregulation of mTOR signaling has been noticed in human malignancies. The emerging data has suggested that the mTOR signal is frequently altered in approximately 30% of cancers.^23,84^ The activation of the mTOR pathway is dependent in three different ways (1) the activating mutations in the *mTOR*, and *mTORC1/2* or mutations in upstream genes lead to hyperactivation of the mTOR signaling(2) overexpression/amplification of the components of mTORC1 and mTORC2 (3) loss of function of negative regulators in the mTOR signaling cascade.^85^ The gain of the function mutations in the kinase domain of mTOR can directly activate the mTOR pathway. The genome sequencing of human tumors has reported approximately 33 mutations in the *mTOR* gene. These mutations were associated with the activation of the mTOR pathway in colorectal cancer, endometroid carcinoma, stomach cancer, lung carcinoma, renal cell carcinoma (RCC), and melanoma.^86^ This study displayed that *mTOR* mutations were clustered in six distinct regions in the c-terminal part of mTOR in human tumors. These mutations did not affect the mTOR complex assembly but suppressed the binding of DEPTOR. Interestingly, the cell lines with *mTOR* mutations displayed marked sensitivity to mTOR inhibitors in both in vitro and murine models.^86^ Moreover, mutations in the components of *mTOR* complexes have been observed in several cancers. For instance, RICTOR was reported to be highly amplified in patients with lung and breast carcinoma. The *RICTOR* amplification in squamous cell lung carcinoma was linked with a bad prognosis and short survival.^87^ Further, this study showed the sensitivity of mTORC1/2 inhibitors against RICTOR-amplified lung cancer cells. Interestingly, the patient was treated with mTORC1/2 inhibitors and displayed stabilization of the tumor for at least 18 months.^87^ Joly et al. also confirmed that RICTOR was robustly expressed in the HER2-amplified breast carcinoma specimens which in turn enhanced phosphorylation of AKT at S473 residue.^88^ A case study by Shamieh and colleagues reported that amplification in the *RICTOR* gene was associated with metastasis and drug resistance in TNBC.^89^ Also, upregulation of RICTOR was associated with increased mTORC2 activity which promoted the cellular motility and proliferation of the glioma cell.^90^ In addition, the hyperactivation of mTOR signaling can be the result of mutations in the upstream genes, including oncogenes and tumor suppressor genes.^34,91^ Gao et al. have reported the overexpression of Rheb1 in acute myeloid leukemia (AML) that was associated with worse median survival. Depletion of Rheb1 in the murine MLL-AF9 model displayed increased survival through suppression of mTOR signaling.^92^ Ghosh et al. reported the mutations in FAT domain of the *mTOR* gene in RCC. This study also identified Rheb mutations in patients with RCC leading to an increase in mTORC1 activity.^93^ The mutations, amplification, and overexpression of *PIK3CA, KRAS, AKT, IGFR*, and *EGFR* are more common in cancer, which are upstream molecular targets for mTOR complexes resulting in the activation of the mTOR signaling cascade in human malignancies. *PIK3CA* mutations are frequently observed in a variety of cancers, including breast, colorectal, and ovarian cancer, and result in the activation of the PI3K/AKT/mTOR cascade. Similarly, mutations in *KRAS*, a key mediator of cell growth and differentiation, can lead to the marked activation of the mTOR signaling and contribute to the process of carcinogenesis. Also, the inactivation of the p53, PTEN, STK11, and TSC1/2 was noticed to enhance the activation of the mTOR signaling cascade in human tumors.^34,94^ The loss of p53 function can contribute to oncogenesis through multiple mechanisms, including promoting cell proliferation and survival, reducing apoptosis, and increasing genomic instability.^95^ The emerging data from various sources have shown that p53 can negatively regulate mTOR signaling by inducing the expression of the mTOR inhibitor, REDD1, and by inhibiting the expression of S6K1, a downstream target of mTOR. In the absence of functional p53, these negative regulatory mechanisms are disrupted, leading to increased activation of the mTOR pathway and promoting tumor growth and survival. Additionally, p53 inactivation can lead to increased expression of growth factors and cytokines, such as IL-6, that can activate the mTOR pathway through other mechanisms. Alterations of PTEN were reported in breast, multiple myeloma, and endometrial cancers, and treatment with mTOR inhibitors displayed strong antitumor activity in these cancers.^96–98^ Inactivation of TSC1/2 has been observed in tuberous sclerosis and can initiate tumorigenesis. The *TSC1/2* mutations have been noticed in many cancers like pancreatic neuroendocrine, urothelial, bladder, and renal.^99–101^ Another side of mTOR signaling is to control the cellular growth and metabolism of the cancer cells through enhanced ribosome biogenesis. Recently, the mTOR complex has been shown as a nutrient sensor in cancer metabolism which includes glucose, lipid, amino acids, nucleotides, growth factors, etc.

### Long noncoding RNAs (lncRNAs) as a regulator of mTOR signaling in cancers

**Fig. 4:**
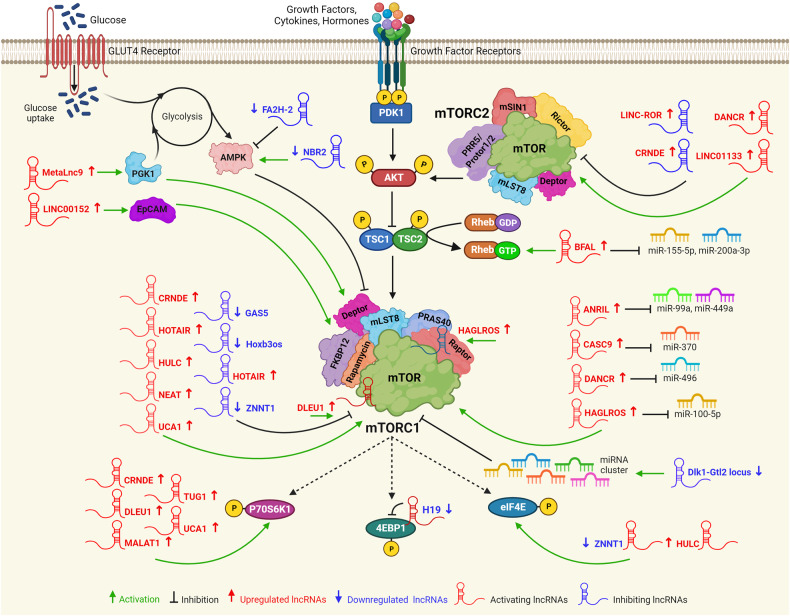
The association of long noncoding RNAs in mTOR signaling. The lncRNAs act as a sponge for miRNA that controls the expression of the upstream or downstream protein coding gene involved in the mTOR signaling cascade. DLEU1 and HAGLROS can directly bind with mTOR whereas FA2H-2 and NBR2 lncRNA regulate mTOR signaling through AMPK. LINC-ROR, CRNDE, DANCR, and LINC01133, regulate mTORC2-mediated signaling. HULC, ZNNT1 activate elF4E, H19 inhibit 4E-BP1, and CRNDE, DLEU1, TUG1, UCA1, and MALAT1 activate P70S6K1 to modulate mTOR signaling. Created with BioRender.com

LncRNAs are >200 nucleotides long RNAs with a close structure of the mRNA that includes 3′-polyadenylated tails, 5′-caps, transcription start site, and splicing resulting in a final gene product (transcript) but do not code for protein.^102–105^ The lncRNAs perform their function by acting as a signal molecule, decoy, guide, and scaffold.^106–109^ During the last decade, a variety of studies have revealed that dysregulated expression of lncRNAs can modulate mTOR signaling and vice versa.^110,111^ The lncRNAs can modulate the mTOR activity in several ways, including (1) direct binding to components of the mTOR complexes and (2) regulating upstream or downstream targets of mTOR. DLEU1 and HAGLROS lncRNAs were reported as direct targets of the mTOR complex through RNA immunoprecipitation.^112^ DLEU1 was overexpressed in endometrial carcinoma than in healthy endometrial.^112^ The overexpression of DLEU1 showed a significant increase in proliferation, clonogenicity, and migration while suppressing apoptosis through the mTOR pathway. Overexpression of DLEU1 resulted in the phosphorylation of mTOR and subsequent activation of downstream targets like PI3K, AKT, and pS70K. This study suggested that DLEU1 enhanced endometrial carcinogenesis through its binding with mTOR protein and activation of the PI3K/AKT/mTOR axis.^112^ Chen and colleagues have noticed that overexpression of HAGLROS was associated with worse outcomes in patients with gastric cancer.^113^ Silencing of HAGLROS suppressed the expression of the mTOR leading to increased expression of ATG9A and ATG9B. Moreover, HAGLROS was found to control mTOR signaling through the sponging of microRNA-100-5p (miR-100-5p) to activate mTOR and its interaction with mTORC1 components to stimulate the mTORC1 pathway.^113^ However, these interactions need to be confirmed through other assays based on RNA/protein crosslinking methods. Several lncRNAs are found to regulate upstream and downstream molecules of the mTOR complex to modulate the mTOR pathway. For example, the NBR2 lncRNA was found to function through the LKB1-AMPK by maintaining the NBR2*-AMPK* feedback-forward loop. Further, the RNA pulldown experiments confirmed the interaction of NBR2 with the AMPK-α subunit. This interaction was markedly enhanced under glucose starvation conditions. NBR2 regulates cell growth, autophagy, and apoptosis in response to energy-related stresses through mTOR signaling.^114^ MALAT1 lncRNA was found to act as oncogenic lncRNA in hepatocellular carcinoma (HCC) via splicing factor SRSF1/mTOR/S6K1 axis.^115^ Overexpression of LINC00152 was reported to increase HCC tumorigenesis regulating the EpCAM expression through mTOR signaling cascade.^116^ Another study displayed that H19 lncRNA was downregulated in human pituitary adenomas. Forced expression of H19 suppressed the cellular proliferation and tumor growth of pituitary cancer cells. Mechanistically, H19 interacts with 4E-BP1 which hampered the 4E-BP1 interaction with Raptor.^117^ This study displayed the potential role of the H19-mTOR-4E-BP1 axis in pituitary tumors.^117,118^ LINC00963 overexpression was associated with poor prognosis, cell proliferation, invasion, and metastasis in non-small cell lung carcinoma (NSCLC). RNA precipitation and mass spectrometry analysis confirmed that LINC00963 interacts with PGK1 which in turn caused activation of AKT/mTOR signaling cascade in NSCLC.^119^ Several groups have displayed that altered lncRNA expression can change mTOR activity or vice versa. The upregulation of the GAS5 lncRNA inhibited the tumorigenesis of gastric carcinoma through the miRNA-106a-5p/AKT/mTOR axis in both in vitro and nude mice xenograft models.^120^ Restoration of the GAS5 expression enhanced the sensitivity of the cisplatin in glioma through mTOR-mediated autophagy.^121^ Depletion of CASC9 suppressed the tumor growth of OSCC xenograft by autophagy-dependent apoptosis via AKT/mTOR pathway.^122^ Silencing of TUG1 lncRNA caused apoptosis of HCC cells through mTOR signaling. Moreover, this study used both the activators and inhibitors of the mTOR/S6K pathway and confirmed that TUG1 controls HCC growth via the mTOR/S6K axis.^123^ CRNDE is one of the highly overexpressed lncRNA in patients with glioma and glioma cell lines. Overexpression of *CRNDE* promoted the growth, clonogenicity, invasion, and migration of glioma cells through increased expression of P70S6K. Mechanistically, the acetylation of histones at the promoter region can lead to the upregulation of CRNDE.^124^ HULC has been found to regulate many cellular processes that are overexpressed in human malignancies. Depletion of *HULC* decreased angiogenesis, proliferation, and invasion by inhibiting the phosphorylation of ERK/AKT/mTOR and downstream target eIF4E.^125^ The UCA1 lncRNA was found to support the increased glycolysis due to the activation of hexokinase 2 via the mTOR-STAT3/miRNA143 axis in bladder cancers.^126^ ZNNT1 lncRNA is localized on chromosome-8 and has only a single exon. ZNNT1 was identified as a downstream target of the mTOR pathway. ZNNT1 expression was induced upon treatment with rapamycin in uveal melanoma.^127^ The overexpression of the ZNNT1 was reported to induce autophagy that regulates tumorigenesis by regulating the expression of ATG12 in uveal melanoma.^127^ We have discussed the role of several other lncRNAs involved in the mTOR signaling in Fig. 4. However, the molecular mechanism(s) behind the deregulation of the lncRNA/mTOR axis need to be investigated in greater detail.

### Role of the mTOR signaling in cancer stem cells

CSCs have been characterized as a unique sub-population that has the ability of self-renewable, metastasis, and drug resistance leading to relapse.^102,128^ During the last decades, CSCs have been observed in a variety of human cancers, including ovarian, pancreatic, breast, liver, and lung.^129,130^ Several groups have displayed the involvement of the Wnt/β-catenin, Hedgehog, STAT3, TGF-β, PI3K/AKT/mTOR signaling cascade in the CSCs. Recently, mTOR signaling has been reported to be critical for CSC self-renewal, maintenance, and tumorigenicity. Zhou et al. have shown the potential role of mTOR signaling in breast cancer stem cells that increased the clonogenic ability and tumor formation in vitro and xenograft models, respectively.^131^ Treatment of HCC with branched-chain amino acids resulted in the activation of mTORC1. Further, the overexpression and silencing approach displayed that mTORC1 activation or silencing of mTORC2 inhibited the CSC population and tumorigenicity by suppressing the expression of EpCAM in HCC.^132^ The activation of the PI3K/AKT pathway was noticed in glioblastoma multiforme (GBM) neurospheres. Interestingly, the combination of alpelisib with pharmacologic mTOR inhibition led to a dramatic and significant decrease in the growth of glioma stem cells.^133^ In another study, the silencing of mTOR or the treatment of rapamycin in A172 cells resulted in the repression of NSC/progenitor markers in GSCs leading to decreased sphere formation. NVP-BEZ235 (PI3K/mTOR inhibitor) treatment demonstrated a significant decrease in the growth of GSCs derived from patient samples in xenograft models.^134^ Importantly, the Yamanaka stem cell factors were used to generate CSCs for understanding the molecular basis of CSCs in human breast cancer cells. This study observed that transcriptional suppression of mTOR repressors plays an important role during the attainment of the CSCs-like characteristics.^135^ This has been reported that PTEN/PI3K pathway is essential for sphere formation and maintenance of CSCs in prostate carcinoma. The NVP-BEZ235 (PI3K/mTOR inhibitor) was effective in suppressing the CSCs and growth of prostate cancer.^136^ The hyperactivation of the PI3K/Akt/mTOR pathway was linked with the upregulation of CXCR4 in A549 gefitinib-resistant (A549-GR) lung cancer cells. This study showed that the population of CXCR4^+^ cells is quite high in the A549-GR cell and has a high capability of self-renewal in vitro and tumorigenicity in the murine model. The CXCR4-mediated STAT3 signaling was also active in A549-GR cells, indicating its importance during the stemness in lung cancer cells.^137^ A study found that phosphorylated IGF-1R was markedly high in breast cancer stem cells (BCSCs) which resulted in mammosphere and tumorigenicity. Interestingly, rapamycin (mTOR inhibitor) displayed a significant reduction in BCSCs in vitro and xenograft model.^138^ Hoshii et al. have generated conditional knockouts of *Raptor*, a component of mTORC1. Depletion of *Raptor* was associated with the inhibition of leukemia in a murine AML model through apoptosis of differentiated leukemia cells. Further, the transplantation of *Raptor*-deficient AML cells demonstrated that mTORC1 is critical for the initiation of leukemia, suggesting that loss of mTORC1 supports the self-renewable ability of leukemic stem cells (LSCs).^139^ Ghosh et al. have reported that depletion of S6K1 enhanced the survival of mice transplanted with MLL-AF9^+^ LSCs through AKT and 4E-BP1 phosphorylation. The S6K1 can work through many targets of the mTOR signaling to increase the renewal and progression of LSCs. The inhibitors against PI3K/mTOR pathway sensitize chronic myeloid leukemia stem cells with tyrosine kinases like nilotinib as well as restore the response of progenitors against nilotinib even in the presence of stem cell factor.^140^ On the other hand, pharmacological inhibition of mTOR was reported with increased expression of CD133 in gastric cancer both in time and dose-dependent fashion.^141^ Yang and colleagues have revealed that suppression of mTOR signaling markedly blocked the conversion of CD133^+^ to CD133^−^ in liver cancer. In xenograft models, the treatment of rapamycin enriched the population of CD133^+^ cells and promoted tumorigenesis of HCC cells.^142^ Altogether, the data from several groups suggested the dual role of mTOR signaling in cancer stem cells in human malignancies. This might be because of different cell types. Therefore, careful thoughts are needed to use mTOR inhibitors against different cancer types.

### Resistance to mTOR inhibitors in human cancers

Drug resistance is one of the most serious problems during the treatment of human cancers in clinics. Several reasons for drug resistance include (1) tumor heterogeneity, (2) clonal selection, (3) evolution of new clones, (4) intrinsic resistance to cell death, (5) complexity and crosstalk among signaling pathways, and adaptation to other survival pathways. The mTOR inhibition revealed promising anticancer efficacy in preclinical models. On the contrary, resistance against mTOR inhibitors has been noticed in several tumors. Efflux of the chemotherapeutic drugs by ABC transporters is one of the essential molecular mechanisms of drug resistance and poor outcomes of treatment. The overexpression of ABC transporters has been noticed in a variety of cancer cell lines treated with mTOR inhibitors. The mTOR inhibitors including AZD8055 and rapamycin were confirmed as a substrate of ABCB1.^143^ NVP-BEZ235 and AZD8055 were found to be transported through ABCG2.^143^ The *Abcb1* and *Abcg2* knockout mice showed enhanced penetration of rapamycin, AZD8055, and NVP-BEZ235 in the brain compared to wild-type mice.^143^ The overexpression of the ABCB1 was associated with resistance to everolimus in luminal breast cancer cells.^144^ NVP-BEZ235 was used in combination with sunitinib against metastatic castration-resistant prostate (mCRPC) cancer and resulted in a synergistic antitumor effect.^145^ The resistance against PF-4989216 PI3K/mTOR inhibitor was observed in lung carcinoma through ABCG2 upregulation. The resistance against PF-4989216 was reversed by inhibition ABCG2.^146^ This was reported that LY3023414 acts as a substrate for both ABCG2 and ABCB1 transporters. The overexpression of ABCG2 and ABCB1 was shown to suppress the intracellular uptake of LY3023414 leading to resistance in cancer cells.^147^ To understand the mechanism of mTOR inhibitors resistance in human cancers, a resistance screen was performed in MCF-7 breast cancer cells and discovered somatic mutations A2034V and F2108L in the FRB-FKBP12 domain of the mTOR to acquire resistance against rapamycin. Also, the M2327I somatic mutation in the kinase domain of the mTOR was observed in the case of an ATP-competitive inhibitor AZD8055.^148^ Importantly, the clinical relevance of somatic mutations has been supported when the F2108L mutation was conferred in the patient who relapsed after the treatment of everolimus in anaplastic thyroid carcinoma.^149^ The mutant tumor cells showed sensitivity to mTOR kinase inhibition. Somatic mutations in the kinase domain including M2327I have been noticed in the drug-naive patients.^86^

## Role of the mTOR signaling in autophagy

**Fig. 5:**
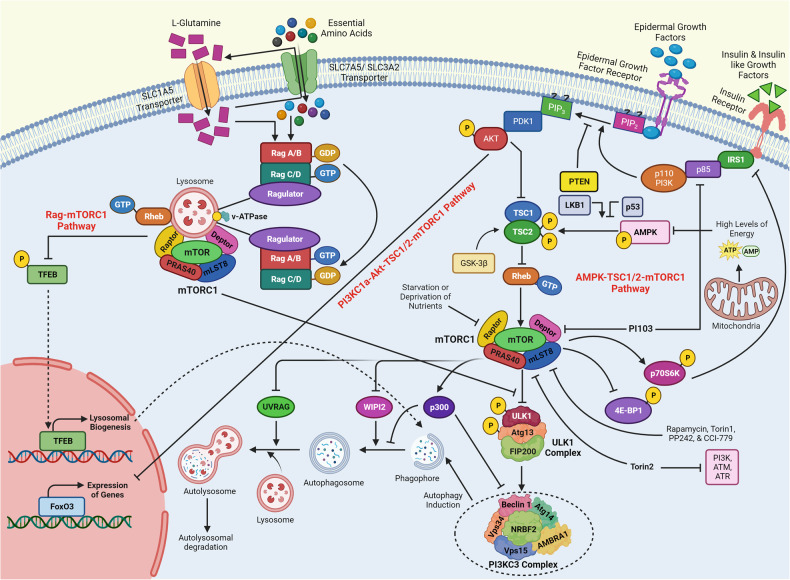
Key events involved in mTOR-mediated autophagy. The mTORC1 suppressed the ULK1 complex activity via phosphorylation of ULK1/ATG13. The mTORC1 can help in the translocation of TFEB in the nuclear compartment to regulate the process of autophagy. The mTORC1 has an important role in the induction of autophagy, nucleation, phagosome elongation, autolysosome formation, and finally degradation through lysosomes. Created with BioRender.com

Autophagy is one of the important processes which are critical for cellular digestion to remove damaged organelles and macromolecules.^150,151^ Apart from this, autophagy is critical in maintaining cellular equilibrium by providing energy and building blocks under stress conditions.^150,151^ Autophagy was reported when the electron microscope revealed the structure of vesicles has amorphous materials and cytoplasmic organelles in the kidneys of newborn murine.^151–153^ Later, studies have demonstrated that the deprivation of amino acid can robustly enhance the process of autophagy perfused livers of rats and mammalian cells.^151,152^ Also, several groups have reported that amino acids are one of the important regulators of the mTORC1 cascade. Under nutrient and growth factor-deprived conditions, mTORC1 activity was reported to be suppressed, indicating that there is an inverse relation between autophagy and activation of mTORC1.^154,155^ The induction of autophagy through inhibition of mTORC1 has been well studied in yeast and drosophila models.^6^ Interestingly, the molecular basis of mTORC1 to regulate autophagy in mammalian cells is quite recent and emerging. mTOR controls autophagy through the regulation of a protein complex composed of UNC-5-like autophagy-activating kinase 1 (ULK1), autophagy-related gene 13 (ATG13), and focal adhesion kinase family-interacting protein of 200 kDa (FIP200). Studies have revealed that mTORC1 can inhibit the ULK complex through the phosphorylation of ATG13 and ULK1/2 (Fig. 5). Silencing of mTORC1 was found to be associated with enhanced activity of ULK1/2 kinase leading to phosphorylation ATG13 and FIP200, the important components of ULK1/2 kinase complex.^156^ The mTORC1 was found to phosphorylate ULK1 at Ser-758 which in turn blocks the interaction with AMPK halting ULK1 activation.^157^ Moreover, mTORC1 was reported to decrease the stability of the ULK1 through phosphorylation of autophagy/beclin 1 regulator 1 (AMBRA1).^158,159^ The mTORC1 and AMPK control the activity of the VPS34 complex which is needed for the generation of the autophagosome.^159^ The ATG14L-associated VPS34 complex has been noticed to play a crucial role in the regulation of autophagy.^160^ Under nutrient stress conditions, AMPK stimulates the autophagy VPS34 complex through phosphorylation of Beclin 1 while suppressing the non-autophagy VPS34 complex via Thr163/Ser165 phosphorylation in VPS34. On the other hand, mTORC1 leads to the phosphorylation of ATG14L in the VPS34 complex to suppress lipid kinase activity of VPS34, suggesting another mechanism for autophagy inhibition through mTORC1.^161^ Moreover, the precise role of autophagy in human malignancies is still not clear because activation or suppression of autophagy was found to be tumorigenic or anti-tumorigenic. Emerging pieces of evidence have shown that activated autophagy can suppress the process of cancer progression, especially in precancerous lesions. However, several studies indicated that autophagy acts to promote tumor survival and growth in advanced cancers.^162,163^ Therefore, the inhibition of autophagy can be employed as a therapeutic approach. Under stress conditions, autophagy supports the growth and survival of tumors, especially in poorly vascularized tumors. Dysregulated autophagy has emerged as an adaptive mechanism for the initiation and progression of human cancers through the accumulation of DNA damage, macromolecules, organelles like mitochondria, oxidative stress, chromatin instability.^164,165^ In addition, stress-induced autophagy has been noticed with enhanced stemness and drug resistance in human cancers.^164,166^

## The importance of mTOR signaling in the aging processes

Several studies have observed that mTOR signaling is involved in the key processes associated with aging in a variety of living organisms, such as worms, yeast, flies, and mammals. Initial studies on the *Caenorhabditis elegans* (*C. elegans*) have noticed that suppressed expression of the mTOR homolog known as ceTOR or let-363 as well as Raptor known as daf-15 was associated with an increased life span of almost more than double.^167^ The mutations in the *CeTOR* and *raptor* were reported with dauer-like larval arrest and suggested that CeTOR is important for the regulation of dauer diapause. The let-363 and daf-15 mutants displayed a marked shift in the metabolism that resulted in fat accumulation and an extended adult life span.^168^ Later on, other genetic screening studies also noticed that reduced mTOR signaling enhanced the life span of the drosophila, yeast, and murine models.^169–171^ Altogether, these studies have proved that extended life span was highly dependent on nutritional condition (glucose, fat, and protein metabolism) and a close association with the mTOR pathway. Interestingly, rapamycin, a pharmacological mTOR inhibitor has been proven to increase the life span in different model organisms.^172–174^ This is well-established that mTORC1 has a major role in nutrients and insulin sensing. Therefore, the benefits of a calorie-restricted diet on life span are because of the reduced mTORC1 signaling. This was confirmed when a calorie-restricted diet did not extend the life span upon inhibition of mTOR signaling in *C. elegans*, Drosophila, and yeast.^170,175^ There are several thoughts regarding the role of mTOR signaling in aging processes in mammalian systems. The repression of the mRNA translation during mTORC1 inhibition was correlated with slower aging by reducing oxidative and proteotoxic stress. This observation was the consistent loss of S6K1 that extends the life span, and resistance to age-related diseases like compromised immunity, motor dysfunction, and loss of insulin sensitivity in mammals.^176^ Also, the loss of S6K1-induced gene expression was similar to a calorie-restricted diet or pharmacological activation of AMPK.^176^ RNA polymerase III (POL III) was reported to play a critical role in nutrient signaling, and anabolic activities to accelerate aging by mTORC1.^177^ There is another possibility that the depletion of mTORC1 could modulate aging via autophagy. Moreover, this was thought that the attenuation of adult stem cells could be central in maintaining aging processes. Rapamycin treatment was found to increase the self-renewal ability of hematopoietic stem cells and the life span of old age mice.^178,179^ Other studies revealed that mTORC1 and SLC7A5 regulate the self-renewal of intestinal stem cell self-renewal and Paneth cell function in maintaining intestinal niche and physiology.^180,181^ The depletion of foxo was found to restore stem cell aging in germline stem cells of drosophila.^182^ Interestingly, the phase IIa clinical trials were conducted on 264 volunteers with an age of ≥65 years of age at 12 clinical sites to evaluate the safety, and efficacy of mTOR inhibitors to boost immune responses.^183,184^ The low dosages of everolimus markedly decreased the rate of infections and enhanced the vaccination response against influenza with increased antiviral immunity.^184^ Based on these data, alternative rapamycin dosage regimens were proposed for better longevity with minimal side effects.^185^ Altogether, mTOR inhibition can increase life expectancy and help delay the onset of age-associated diseases. However, the duration of the treatment should be decided carefully as it can produce severe side effects like immunosuppression and glucose intolerance.

## mTOR signaling in neurological processes, brain development, and neurological diseases

The emerging research studies displayed that mTOR signaling is one of the important regulators of neurological processes like neural stem cells, neural development, synaptic plasticity, circuit formation, learning, and memory.^186^ Ablation of Rictor or Raptor in neurons was found to decrease the neuron size and early death.^187^ The depletion of Rictor or Raptor was shown to have a differential impact on the differentiation of the oligodendrocyte and myelination of the central nervous system. These data revealed the importance of mTORC1 and mTORC2 during brain development.^187^ On the contrary, the hyperactivation of the mTORC1 pathway was observed in the brain and well-documented in human patients with TSC. These patients have been shown with several neurological disorders such as autism, intellectual disability, epilepsy, anxiety, sleep disturbances, and brain tumors.^188,189^ TSC has been characterized as an autosomal disorder caused by the loss of either TSC1 or TSC2. Moreover, the hyperactivation of mTORC1 because of the *Tsc1* or *Tsc2* loss in the neural cells in the murine models displayed severe epileptic seizures. The rapamycin treatment was effective in reducing epileptic seizures in these mice.^190^ The mutations in components of the KICSTOR and GATOR1 complexes were associated with epilepsy in humans. Recently, the retrospective analysis of TSC patients treated with everolimus or sirolimus under the age of 2 years has reported promising benefits on epilepsy.^191^ This study for the basis for the testing of mTOR inhibition in a large cohort of patients with TSC. The mTORC1 activation in tissue stems promoted mRNA translation near synapses which required neuronal circuit formation. Further, inhibition of mTOR signaling was found to block the ketamine-induced synaptogenesis and behavioral responses.^192^ The mTORC1-mediated autophagy was found to be strongly correlated with the pathogenesis of neurodegenerative disorders like Alzheimer’s disease (AD), and Parkinson’s disease (PD). The rapamycin treatment was found to inhibit the progression of AD and increase the life span in the diseased model of AD.^193^ Rapamycin is used in clinical settings and has shown promising effectiveness against AD and emerged as a potential therapeutics.^194^ Dactolisib treatment was shown to protect the AD in a transgenic murine Alzheimer model.^195^ In the future, next-generation mTOR inhibitors can be designed and tested in AD and PD mice models for better drugs.

## Role of mTOR signaling in maintaining the immune response

**Fig. 6:**
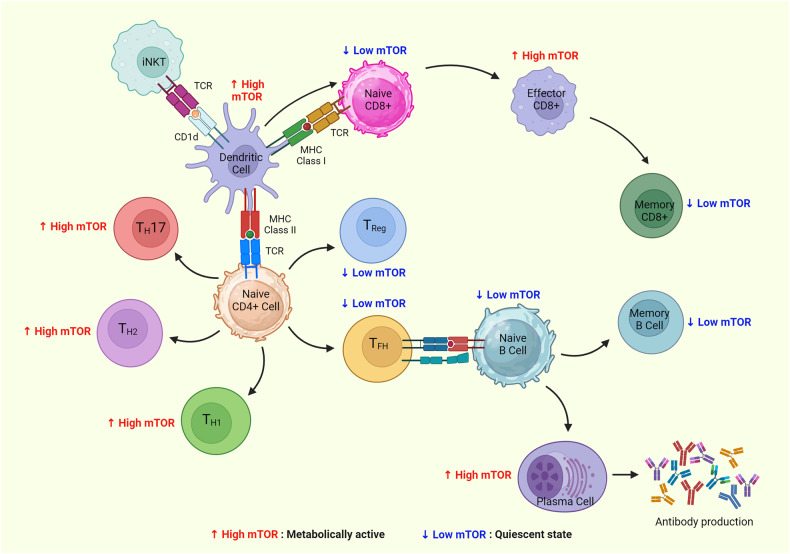
The mTOR signaling is crucial for modulating the key immune responses. The activated dendritic cells can present the antigen through T-cell receptors that initiate activation, proliferation, and differentiation of invariant natural killer T cells, CD4^+^, and CD8^+^ T cells through stimulation of mTOR. The higher levels of mTOR determine the metabolic active state while lower levels of mTOR define the quiescent state of the T- and B cells. The absence of mTOR signaling during differentiation of naive CD4^+^ T cells generates T-regulatory cell and T-follicular helper cell. The high mTOR activity during activation of naive CD4^+^ T cells supports the expression of the crucial transcription factors needed for their differentiation into Th1, Th2, and Th17 cells. T-follicular helper cells activate the germinal center to generate antibodies. Created with BioRender.com

Recent reports have uncovered a significant regulatory function of the mTOR pathway not only in cancer but also in the differentiation, activation, and functional characteristics of immune cells.^196^ Tumors can evade the immune system by dampening its ability to detect and eliminate cancer cells.^197^ Recent research has focused on tumor immunotherapy as a promising approach to tackle this challenge. Multiple studies indicate that the mTOR pathway, which is frequently overactive in tumors, plays a role in controlling the development and effectiveness of immune cells.^5^ Moreover, mTOR signaling plays a vital role in enabling T cells to perceive and merge immune signals originating from dendritic cells (DCs).^198^ These signals have been found to involve cytokines, co-stimulatory molecules, and antigenic signals as well as environmental cues derived nutrients, growth, and immunoregulatory factors.^198^ T cells rely on mTOR signaling to sense and integrate this comprehensive array of immune and environmental inputs. Studies have identified that TSC1 acts as most crucial for maintaining the naive T-cell quiescence and survival to maintain immune homeostasis.^199,200^ The T cells that were deficient for PTEN were reported to upregulate mTORC1 activity to maintain their quiescence before tumorigenesis indicating the importance of mTORC1 on T-cell homeostasis.^201^ Other studies have displayed that phosphorylation of liver kinase B1 or STK11 phosphorylates stimulates AMPK under energy deprivation. Further, deletion of *Lkb1* in T cells caused massive T-cell apoptosis while compromising thymic selection, altered metabolism, and proliferation of T cells.^202,203^ The stimulation of T- and B-cell receptors as well as other cytokine receptors, such as the IL-2 receptor, causes mTOR to become active in the adaptive immune system. In cytokine-stimulated T cells, mTOR regulates the transition from the G1 to the S phase of the cell cycle.^204,205^ In addition, once T cells are activated by IL-12, mTOR may drive the development of Th1 cells by stimulating the production of IFN-ϒ.^206^ Further, studies have reported that the antigen recognition by naive T cells leads to the mTOR activation, which guides the differentiation of CD4^+^ T cells into the T-helper cell effector lineages. Also, mTOR was shown to regulate the effector fate of CD8^+^ T cells during tumor immunity and infections.^207^ Interestingly, several studies have primarily focused on mTOR inhibitor rapamycin’s ability to hinder T-cell proliferation and IL-2 production and induce anergy (a cellular state when the lymphocytes fail to respond upon stimulation), even in adequately stimulated T cells.^196,198,205^ Upon T-cell receptor (TCR) stimulation, both mTORC1 and mTORC2 are activated. The mTORC1 was found to influence the effector responses of T cell (CD8 +) whereas mTORC2 activity was associated with metabolic reprogramming to generate memory T cell (CD8 +).^208^ The extent of mTOR activation is directly linked to the duration of the interaction between T cells and DCs, as well as the amount of the corresponding antigen (Fig. 6). In addition, co-stimulatory signals exert a significant influence on mTOR activity. CD28-mediated co-stimulation, a well-known activating signal for the PI3K–AKT pathway, enhances the mTOR activity induced by TCR stimulation. This synergistic effect facilitates effective T-cell activation by upregulating mTOR function. The role of mTOR signaling in B-cell development, differentiation, and function is less studied than in T-cell development. The mTOR hypomorph mouse model was generated by *neo*-insertion which partially disrupts *mTOR* transcription. This murine model displayed a partial block of large pre-B to small pre-B stages of B-cell development and compromised proliferation in response to B-cell mitogenic signals. B-cell receptor (BCR) and CD40 signaling were more highly compromised than TLR signaling.^209,210^ This resulted in the alteration in the splenic populations, production of antibodies, and migration towards chemokines.^209^ The deficiency of SIN1 resulted in the increased expression of IL-7 receptor (il7r), rag1, and rag2 which is responsible for increased V(D)J recombinase activity and survival of the pro-B-cell. This study showed that Akt2 mediates the Sin1-mTORC2-dependent inhibition of il7r and rag gene expression which in turn control the phosphorylation of FoxO1 during B-cell development. Interestingly, mTOR inhibition using rapamycin enhanced rag expression and V(D)J recombination in B cells. This study indicated Sin1/mTORC2-Akt2/FoxO1 axis is critical in B cells.^211^ *Rictor*^fl/fl^*Mx1*-Cre mice displayed an increase in the population of pro-B, pre-B, and immature B cells with a significant decrease in mature B cells.^212^ The deficiency of *Rictor* in B cell resulted in the upregulation of *IL-7R*, *RAG1*, and decreased phosphorylation of Foxo1.^212^In the absence of T-cell antigens, the mTOR signaling can regulate antigen titration which regulates B-cell activation. Also, the activation of mTOR signaling by BCR stimulation and repression of the mTOR pathway by Fc receptor signaling exhibited the crucial role of mTOR in regulating B-cell functions.^213^ Raptor was specifically deleted in the murine B cell by crossing *Raptor*^fl/fl^ mice with *Mb1*-Cre transgenic mice. *Raptor*^fl/fl^*Mb1*-Cre model demonstrated the block in the early pre-B-cell stage with the loss of immature and mature peripheral B cells. *Raptor-deficient* pre-B cells were found to have reduced survival, proliferation, oxidative, and glycolytic metabolic capacity.^214^ Interestingly, the treatment of these mice with Rapamycin recapitulated the early block during B-cell development, and loss of immature B cells with the accumulation of pre-B cells.^214^ Recently, transcriptomic analysis of the B cells displayed that follicular B cells upregulate a network of unfolded protein response genes before the secretion of antibodies. The transcription of unfolded protein response genes needs Raptor and mTORC1 kinase adapter. This study suggested that B cells exploit mTORC1 for subsequent plasma cell function and prior antibody production without Xbp1 activity.^215^

Growth factors, TLR ligands, cytokines, and other extracellular signals can all activate the mTORC1-mTORC2 network in innate immune cells.^216^ Studies have demonstrated that mTOR signaling also plays a crucial role in the differentiation and function of dendritic cells (DCs) and natural killer (NK) cells.^217^ DCs possess strong antigen presentation abilities, and NK cells are important immune cells involved in tumor surveillance.^218–220^ Previous studies have reported that both mTORC1 and mTORC2 exert distinct effects on NK cell function.^221^ While mTORC2 negatively regulates NK cell function by inhibiting the STAT5/SLC7A5 axis, mTORC1 positively regulates mTORC2 activity, thereby promoting the CD122-mediated interleukin-15 signaling pathway.^222^ The cytokine interleukin-15 (IL-15) induces mTOR activity in NK cells in both humans and mice. Additionally, recent studies have linked DCs to the mTOR signaling pathway. Inhibition of mTOR in DCs has been shown to improve their antigen presentation capabilities and enhance the activation of cytotoxic CD8 + T lymphocytes, resulting in increased antitumor activity.^223,224^ Therefore, mTOR inhibitors hold promise for enhancing the efficacy of tumor immunotherapy and autologous DC-based vaccination by extending the life span of DCs and improving their antigen-processing abilities.

Macrophages, specifically the M1 and M2 subtypes, play critical roles in tumor development and progression.^225,226^ M1 macrophages can kill tumor cells, while M2 macrophages promote tumor growth, invasion, and metastasis.^227^ Dysregulation of the mTOR pathway has been implicated in the polarization and function of macrophages. For instance, decreased expression of miR-30c, which inhibits mTOR activity, leads to the inhibition of M1 macrophage differentiation and function, ultimately promoting tumor growth and metastasis.^5^ These findings highlight the intricate relationship between the mTOR pathway and macrophage polarization, shedding light on potential therapeutic strategies to modulate immune responses in the tumor microenvironment.^196^ Studies have shown that enhanced PI3K and mTOR signaling in mouse macrophages leads to increased M2 macrophage markers and activation of the STAT6 pathway, which promotes M2 polarization.^228^ Conversely, inhibiting mTORC1 in human macrophages enhances M1 polarization. However, the role of mTORC1 and mTORC2 can be complex, as the deletion of Tsc1, a component of mTORC1, can promote both M1 and M2 polarization depending on different pathways.^229^ AKT, another protein in the pathway, also has isoform-specific effects on macrophage polarization. The PI3K–AKT–mTOR pathway is involved in sensing these cues and affecting macrophage polarization, although the mechanisms are not fully understood. Taken together, mTOR inhibition has enormous potential for vaccine development to boost immunity against human malignancies and pathogens.

## Opportunities and challenges of mTOR signaling in cancer therapeutic targeting

**Fig. 7:**
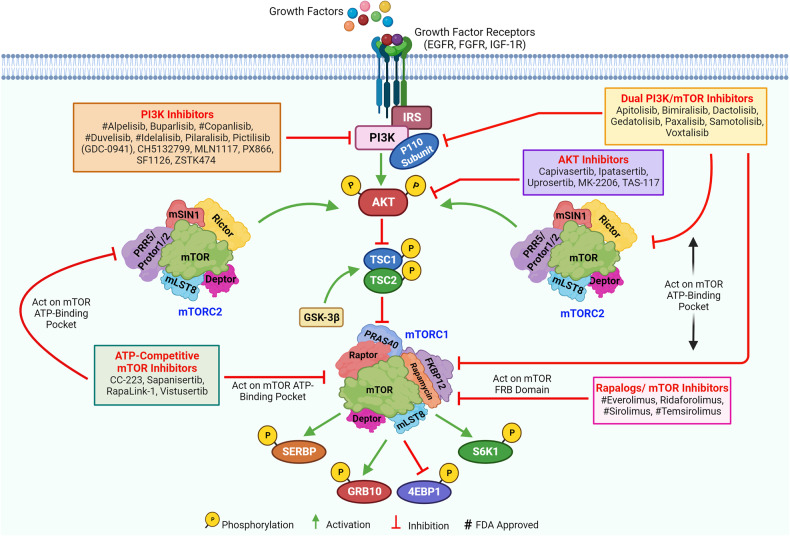
Pharmacological targeting of the mTOR signaling cascade in human malignancies. The mTOR pathway can be targeted at different levels in human malignancies using Rapalogs or mTOR inhibitors, PI3K small molecule inhibitors, dual mTOR/PI3K inhibitors, compounds targeting AKT, and ATP-competitive inhibitors against mTOR. Created with BioRender.com

Rapamycin is a natural macrocyclic lactone that is obtained from the bacterium *Streptomyces* *hygroscopicus*.^230,231^ After the discovery of rapamycin, researchers continued to explore the related targets and signaling involved at the molecular and cellular levels.^230,232^ The FKBP12 and FKBP51 expression was found to be the rate-limiting factor that decides the rapamycin drug response in cell lines and tissues.^233^ This led to the discovery and development of various moieties including synthetic/semisynthetic which were active against mTOR-induced oncogenesis.^232,234,235^ Sirolimus is another approved biochemical functional form of rapamycin that displayed target-specific inhibition.^236^ Sirolimus has been found to impair the molecular interaction between mTOR and Raptor by targeting mTORC1.^234,237,238^ Sirolimus is an oral drug that has high protein binding, an elimination half-life of 57–63 h, high drug distribution to the circulation, and is metabolized by the hepatic enzymes like CYP3A4, CYP3A5, and excretion through p-glycoprotein.^239^ Sirolimus was approved by FDA for organ transplantation as an immunosuppressive agent in the year 2009. Interestingly, therapeutic drug monitoring was found to be a critical aspect during sirolimus treatment.^240^ Siromimus received FDA approval in the year 2015 for lymphangioleiomyomatosis based on the efficacy reported in clinical trials (ClinicalTrials.gov number, NCT00457808, and NCT00414648).^241,242^ Nab-sirolimus (albumin-bound nanoparticles-based sirolimus formulation) displayed a strong antitumor effect in cases with perivascular epithelioid cell tumors (PEComa). Nab-sirolimus was finally approved by FDA after the success of the AMPECT phase-II trials against metastatic or unresectable PEComa in 2021.^243^ Recently, Nab-sirolimus is going through a clinical trial in solid tumors with genetic mutations in *TSC1* and *TSC2*.^244^The poor bioavailability of the drug led researchers to focus mainly on improving its pharmacokinetics and stability without disturbing its pharmacodynamic profile.^245^ Two sides are obligatory to interact with FKBP12 and mTOR providing few options for structural modifications. Synthetic analogs or derivatives thus designed, synthesized, and biologically evaluated by replacing the hydrogen of –OH (C-40) with different moieties led to the development of several newer analogs.^246^ These analogs have been named Rapalogs with improved pharmacokinetics and stability without disturbing their pharmacodynamic profile. Temsirolimus (a rapalog) was designed by replacing H of the hydroxyl group (C-40) with di-hydroxyl methyl propionic acid ester and was finally authorized by the FDA for the treatment of the patients with metastatic RCC in the year 2007 due to very high bioavailability, specificity, long half-life, good excretion through fecal and urine.^247–250^ Temsirolimus is well-characterized pharmacological derivative of sirolimus. Temsirolimus was reported to interact with FKBP12 that resulted in the formation of a strong trimolecular complex with mTOR. Temsirolimus was found to be equivalent to sirolimus in terms of its inhibitory activity against mTOR kinase both in the cellular and cell-free systems.^251^ Temsirolimus was noticed with remarkable antitumor potential in human preclinical and clinical trials for cancers as a single agent (monotherapy) or in combination with other chemotherapeutic agents in refractory/relapsed acute lymphocytic leukemia, cervix, endometrial and ovarian cancers.^252–256^ Everolimus was designed as an immunosuppressive drug by replacing H of the hydroxyl group (C-40) with a hydroxylethyl group.^257^ Everolimus is an orally administered drug that possesses high protein binding, and a good half-life, is metabolized mainly through CYP3A enzyme, has good blood-brain penetration, and is eliminated by feces and urine. This has been observed that both temsirolimus and everolimus demonstrated anticancer activity in murine models.^9^ Importantly, these are commonly used chemotherapeutic agents for treating advanced-stage RCC in the clinic. Everolimus is used for patients with advanced breast carcinoma or neuroendocrine pancreatic carcinoma.^258–260^ The combination of exemestane to everolimus markedly enhanced the progression-free survival in HR(+) breast carcinoma and reduced recurrence in breast cancer trials of oral EveROlimus-2 (ClinicalTrials.gov; NCT00863655). The combination of everolimus with sunitinib/AZD2014 was not tolerated in the cases with metastatic RCC during clinical trials suggesting that the combination of two parallel signalings should be thought carefully.^261–263^ The everolimus was found to be effective in clinical phase-II trials in patients with advanced thyroid carcinoma with low toxicity and further trials are needed using combination therapy.^264^ Recently, everolimus was used combined with T-DM1 which resulted in marked antitumor efficacy in HER2-positive breast cancer, suggesting the importance of mTOR-dependent lysosomal processing of T-DM1.^265^ Everolimus and letrozole were correlated with a promising progression-free survival till 12 weeks of ER^+^ relapsed high-grade ovarian carcinoma in phase-II clinical trials.^266^ Everolimus was used in combination with rituximab and reported complete responses in patients with relapsed diffuse large B-cell lymphoma (DLBCL) under NCT00869999 (ClinicalTrials.gov).^267^ Everolimus treatment has been found to reduce the size of subependymal giant-cell astrocytomas and the frequency of seizures (ClinicalTrials.gov, NCT00411619) in these patients.^268^ Everolimus has reported promising anticancer efficacy in patients with pancreatic neuroendocrine tumors during clinical trials (RADIANT-3, NCT00510068) and received FDA approval.^269^ Everolimus was given approval by the FDA in the year 2016 for clinical use in patients with neuroendocrine tumors of the gastrointestinal tract or lung origin.^270^ Ridaforolimus is the FDA-approved small molecule inhibitor of mTOR(C-40 phosphine oxide substituted formulation of rapamycin) which is available orally or intravenously for the treatment of human soft tissue and bone sarcoma in phase-II clinical trials (ClinicalTrials.gov; NCT00093080) and results showed remarkable efficacy and safety.^271–273^ The results of the phase-III international randomized clinical trial of ridaforolimus (NCT00538239) displayed tumor regression in the patients with metastatic sarcomas compared to the placebo control group.^272,274^ Ridaforolimus displayed potent antitumor activity in advanced endometrial cancer with significant side effects and represent a viable therapeutic agent for PI3K/Akt/mTOR pathway in trastuzumab-resistant and HER2-positive metastatic breast carcinoma and hematological malignancies.^273–277^ The safety and efficacy profiling of the vistusertib/ AZD2014 was evaluated in a variety of preclinical and clinical studies against human cancers.^278,279^ Further, the AZD8055 is another very specific and highly potent dual mTORC1/2 inhibitor with superior anticancer effect in a variety of human malignancies.^280,281^ In the case of leukemia, the AZD8055 treatment displayed enhanced prolonged survival of AML transplanted mice while suppressing the growth of the leukemic cells with managed toxicity profile.^282–284^ These rapalogs were also evaluated for their anticancer activity for the treatment of advanced-stage cancers including liver, gastric, endometrial, lung, and mantle cell lymphoma.^7,9^ Rapalogs have been reported to block mTORC1 substrates selectively and effectively. mTORC1 inhibition and S6K1-dependent pathway inhibition by rapalogs were reported to result in feedback activation of PI3K, Ras/MAPK, mTORC2, RTK’s, and AGC kinases.^285^ Feedback activation of these proteins and pathways was found to compromise the mTORC1 inhibition in disease stabilization, protein biosynthesis, and cell cycle contributing to the development of resistance to rapalogs. O’Reilly et al. found that sirolimus therapy augments AKT phosphorylation at Ser473 residue. The phosphorylation of AKT was also prominent in tumors from patients receiving everolimus as a part of treatment. On the contrary, this has been observed that despite disrupting the S6K-IRS-1-negative feedback loop, sirolimus therapy was potent in preventing carcinogenesis. Also, the genetic variations of the factors associated with the mTOR signaling pathway in cancer cells contribute to de novo resistance to the drugs targeting mTOR.^286^ A twist in the mTOR story has emerged with the finding that mTORC2 can directly phosphorylate AKT and stimulate a downstream signaling cascade. Unexpectedly, it was noticed that rapamycin can inhibit AKT by disrupting mTORC2 assembly in a few types of cells.^287^ These discoveries raise many questions regarding the development and application of mTOR inhibitors. To overcome these limitations new strategies were designed and explored. These strategies included combination therapy which includes rapalogs in combination with chemotherapeutic drugs such as paclitaxel or carboplatin and the development of inhibitors that can target both mTOR and PI3K.^9,288–291^ Pharmaceutical companies and academic laboratories have developed numerous such inhibitors by exploiting the structure of the ATP binding pocket of kinases and the small ATP-competitive molecules. Because of sequence similarity among the PI3K and mTOR, several PI3K inhibitors were found to inhibit mTOR activities. These were called dual PI3K/mTOR inhibitors^289^ and displayed promising activity to overcome the negative feedback associated with resistance of rapalogs. Because of the diverse functions of different isoforms of PI3K, these dual inhibitors were not well tolerated owing to potential toxicities. These drawbacks fueled the development of inhibitors with higher mTOR specificity than PI3K.^292^ Researchers then focused on designing such inhibitors that inquest the development of a series of such specific inhibitors. Preclinical and early clinical studies revealed that TOR-KI inhibitors can stop the cell cycle in the G1 phase and suppress the transcription of cyclin D1, which is mTORC1 dependent and activated by AKT.^9,293^ These inhibitors also inhibit the activity of Akt and SGK1, which phosphorylate p27, leading to better inhibition of cyclin-CDK2. By inhibiting the production of HIF-2α, TOR-KI inhibitors prevent RTK accumulation and growth factor independence, which is favored by HIF-1 and 2α leading to autonomous growth.^1,9^ Irrespective of the good preclinical and early clinical status of TOR-KI, it was observed by the researchers that the TOR-KI treated cells attenuated mTORC2-mediated phosphorylation of AKT at S473 residue but mTORC1 inhibition can still promote the feedback activation of PDK1 and PI3K derived AKT phosphorylation at T308. This suggested the modest substrate dependency of AKT phosphorylation specifically at S473 residue.^294^ Although the TOR-KI efficiently inhibited the activity of both TOR complexes but are still quite ineffective because of various feedback loops contributing through upstream signaling pathways as well as the wide range of clinically relevant mutations in mTOR.^295^ Mutations increase the catalytic activity of mTORC1/2 and thereby reduce the effectiveness of such compounds towards rapalogs, dual inhibitors as well as TOR-KI. To overcome these resistance issues Rodrik-outmezguine and colleagues have generated rapamycin-resistant breast carcinoma cell lines that carry two mutations in the mTOR FRB domain (mTOR A2034V and mTOR F2108L).^148^ They also generated the AZD8055-resistant colony that bears mutations in the hyperactive kinase domain. A careful study of the molecular model of mTOR was done revealing the juxtaposition of rapamycin and AZD8055 binding sites. The knowledge of these binding sites has provided the basis for designing new small molecules which finally resulted in a new bivalent mTOR inhibitor.^7,286^ The molecules were designed by linking rapalogs and TOR-KIs using an optimum-length cross-linker. Preclinical studies were performed on murine xenografts of MCF-7 cells bearing these mutations and have reported more sensitivity to rapalink as compared with rapalog and TOR-Kis. This was because of the rapalink(s) capability to inhibit both mTORC1/2, whereas rapamycin and dual inhibitors were not able to block the activities of both complexes effectively even at higher concentrations.^148^ Rapalinks are third-generation mTOR inhibitors with advanced structural features of first and second-generation inhibitors that overcome several issues like efficacy, resistance, and feedback activation because rapalink can efficiently bind with the FRB domain of mTOR through binding to FKBP12 and kinase domain of mTOR for its ATP-competitive inhibition simultaneously.^296^ Sapanisertib (MLN0128 or TAK-228), a selective inhibitor of mTOR reported promising anticancer effects during preclinical analysis. After preclinical evaluation, sapanisertib entered phase-I/II clinical trials for mCRPC, breast carcinoma, lung cancer, endometrial carcinoma, bladder carcinoma, sarcoma, and non-Hodgkin B-cell lymphoma.^297–304^ MLN0128 treatment was found to be very effective in suppressing the tumorogenesis of different subtypes of sarcoma in both in vitro and xenograft models.^297^ MLN0128 was effective in sensitizing the chemoresistant primary effusion lymphoma.^305^ Studies have shown that MLN0128 suppresses the growth of HCC tumors and sensitizes them to sorafenib and cabozantinib.^303,306,307^ Interestingly, MLN0128 was effective in sensitizing the everolimus-resistant *PIK3CA* mutant colorectal and pancreatic neuroendocrine tumors.^308,309^ Recently, the DICE trial revealed that TAK-228 along with weekly paclitaxel chemotherapy resulted in significant improvement and laid the foundation for a phase-III trial in ovarian cancer.^310^ The CC-223 is an oral and potent mTOR kinase inhibitor that was found to suppress the growth and tumorigenesis of HNSCC and HCC through mTORC1/2 in both in vitro and murine model.^311–314^ The preclinical studies displayed that OSI-027 is a selective and potent inhibitor of mTORC1/2 and reported promising antitumor activity in several human cancers.^315–318^ Further, OSI-027 treatment was very effective in enhancing the therapeutic potential of gemcitabine in pancreatic cancer cells and xenograft model.^319,320^ The dual PI3K/mTOR inhibition has emerged as a critical strategy to target mTOR signaling in human malignancies.^321^ For instance, NVP-BEZ235 (dactolisib) was shown to suppress the PI3K isoforms, mTOR, and ATR which resulted in strong anticancer activity in both solid and blood cancers.^322–324^ Also, NVP-BEZ235 was displayed to cross the blood-brain barrier and reported to sensitize the temozolomide resistance in brain tumors.^325^ Strikingly, the combination of dactolisib with immune checkpoint inhibition in primary and metastatic CRPC displayed robust antitumor response by blocking MDSCs.^326^ The 17AAG (HSP90 inhibitor) was used in combination with NVP-BEZ235 and resulted in synergistic anticancer activity in melanoma by targeting both MAPK and PI3K/AKT/mTOR signaling pathways.^327^ Similarly, paxalisib, apitolisib, voxtalisib, PQR309, XH00230381967, omipalisib, and gedatolisib were validated as potent PI3K/mTOR inhibitors with potent antitumor efficacy in many human cancers.^9,328–330^ Paxalisib either as a monotherapy or in combination with ONC201 has entered the phase-II (NCT05009992) clinical trial for the diffuse midline glioma.^331–334^ Apitolisib is an oral and potent inhibitor of mTORC1/2 and class I PI3K. Many preclinical studies have tested the activity of GDC-0980 in solid tumors including lung, brain, endometrial, and gall bladder.^335,336^ Phase-I clinical trials were conducted to evaluate the tolerability, safety, and antitumor efficacy of GDC-0980 in solid tumors.^337^ Voxtalisib was used at a concentration of 50 mg BID and reported promising anticancer activity in patients with refractory/relapsed follicular lymphoma as well as limited efficacy in DLBCL, chronic myeloid leukemia, and mental cell lymphoma during an open-label, phase 2 trial (ClinicalTrials.gov; NCT01403636) and solid tumors.^328,338^ The combinatorial effect of the voxtalisib with low intensity pulsed ultrasound displayed to inhibit GBM tumorigenesis while suppressing the PI3K/AKT/mTOR signaling in CSCs.^339^ Another study showed the synergistic effect of the voxtalisib when used either with temozolomide plus radiotherapy in glioma.^340,341^ PQR309 was developed as a dual PI3K/mTOR Inhibitor and was found to have marked antitumor efficacy in lymphomas as a monotherapy and in combination with venetoclax, lenalidomide, ibrutinib, panobinostat, and rituximab.^329^ PQR309 has been reported with promising efficacy against solid and blood cancers.^279,342,343^ LY3023414 (PI3K/mTOR and DNA-PK inhibitor) was reported with anticancer activity alone and in combination with clinically approved chemotherapeutic drugs in several cancers.^344,345^ Du and colleagues have reported that omipalisib acts as a sensitizer for DNA damage-induced apoptosis of Hela cells through suppression of non-homologous end joining pathway (NHEJ). Further, the combination of omipalisib and doxorubicin-induced γH2AX in A549 cells. Omipalisib was found to inhibit DNA-PKcs kinase activity in a dose-dependent fashion indicating the ability of omipalisib to directly suppress DNA-PK (a core component in the NHEJ pathway).^346,347^ Interestingly, the gedatolisib was found to be potent to increase the efficacy of the radiotherapy HNSCC and nasopharyngeal carcinoma indicating that gedatolisib may be utilized as a sensitizer to radiotherapy.^348,349^ The phase-I study using gedatolisib in combination with carboplatin and paclitaxel revealed 80% response rate with acceptable tolerability clear cell ovarian carcinoma.^350^ Recently, phase 1 and 1b studies used the combination of gedatolisib plus cofetuzumab pelidotin and revealed promising clinical anticancer response and tolerable toxicities in patients with metastatic TNBC, endometrial and other advanced cancers.^351–357^ Metformin has been shown to activate AMPK through repression of mitochondrial respiratory chain complex I leading to an increase in the AMP/ATP ratio.^358^ Mechanistically, AMPK negatively regulates the activity of the mTORC1. Wang and colleagues have reported that metformin suppressed mTORC1/2 through the activation of AMPK in myeloma cells. Moreover, metformin suppressed the tumor formation in the mouse model of myeloma through the overexpression of AMPK and downregulation of mTOR.^359^ In addition, metformin was reported to silence mTORC1 through Rag GTPases/REDD1 axis which is independent of AMPK signaling. Metformin was reported to enhance autophagy through AMPK stimulation and mTORC1 inhibition.^359^ Metformin treatment displayed strong anticancer efficacy in human malignancies including breast cancer through repression of mTORC1-dependent protein synthesis.^360^ Recently, the application of metformin in the context to autophagy has been manifested in preclinical models against human cancers. Pharmacological targeting of AKT/PI3K/mTOR axis has been summarized in Fig. 7 and Table 1.

**Table 1:** mTOR and dual PI3K/mTOR inhibitors in preclinical studies and clinical trials against human malignancies

Name of the drug/inhibitor (s)	Mechanism of action/target (s)	Disease/condition(s)	Status of the clinical phase trial(s) and Identifier(s)	Advantage(s)	Limitation(s)	IC_50_ in cancer cell lines	Reference (s)
mTOR Inhibitors							
Sirolimus (Rapamune)	Interact with FKBP12 to form a complex that suppressed mTORC1 activity.	Organ transplants. Lymphangioleiomyomatosis Relapsed/Refractory Lymphoblastic Leukemia and Lymphoma Relapsed and Refractory Solid Tumors HNSCC	FDA-approved in the years 1999 & 2000 FDA-approved in the year 2015 NCT00414648, Year-2006 NCT01162551, Phase II, Year-2010 NCT01658007, Phase I, Year-2012 NCT01670175, Phase I, Year-2012 NCT01195922, Year-2010	Potent, promising, and well-established drug with good clinical efficacy and safety profile. Enhanced radiosensitivity of HNSCC.	Limited solubility and bioavailability with a low toxicity profile	Human OS cell line MG63/ADM = 23.97 nmol/L Murine leukemia cells (P388) = 0.1 nmol T-cell line = 0.05 nmol	^234–242^
Nab-sirolimus	Bind with FKBP12 to form a complex that suppressed mTORC1 activity.	Advanced unresectable/ metastatic malignant PEComa. Malignant solid tumors harboring inactivating alterations in *TSC1* or *TSC2*.	Nab-sirolimus was FDA-approved through AMPECT trial number NCT02494570 in the year 2021. NCT05103358, Phase II, Year-2022	The ORR of the drug was highly promising, and 2 patients showed a complete response.	Fatigue, stomatitis, rash, nausea, musculoskeletal pain, infection, edema, diarrhea, vomiting, loss of weight and appetite,		^243,244^
Temsirolimus (Torisel, CCI-779)	FKBP12 subunit of mTORC1	Renal cell carcinoma Relapsed/refractory ALL, recurrent ovarian, endometrial cancer, and mCRPC	FDA was approved in year-2007 NCT01403415, Phase I, Year 2011 NCT01614197, Phase I, Year-2012 NCT01083368, Phase I/II, Year 2009	Promising anticancer efficacy, high bioavailability, specificity, the long half-life for convenient weekly dosing, good excretion through fecal and urine	Adverse effects, including rash, asthenia, anemia, mucositis, hyperlipidemia, and hyperglycemia	Renal cell carcinoma cells (786-O) = 5 Hep3B = 210 HepG2 = 190 HuH7 = 0.22 PLC = 0.4	^247–256^
Everolimus (Afinitor, RAD001)	FKBP12 subunit of mTORC1	RCC, neuroendocrine tumors of GI or lung origin, PNET, TSC-associated diseases. SEGA Pediatric ALL	Everolimus received FDA approval in 2009 for RCC, in 2011 for PNET, in 2012 for breast carcinoma, in 2016 for neuroendocrine tumors, 2018 for TSC-associated diseases. NCT00411619, Year-2007 NCT01523977, Phase I, Year-2012	The broad range of clinical applications with enhanced efficacy and prolonged on-target effects	Adverse effects, such as immunosuppression and metabolic disturbances	Renal cell carcinoma cells including 786-O = 1.8–2.6 Breast cancer cell lines include MDA‐MB‐231 = > 200 MDA-MB-468 = 0.7 BT549 = 1.6	^257–270^
Ridaforolimus (Deforolimus, AP23573, MK-8669)	FKBP12 subunit of mTORC1	Metastatic soft-tissue and bone sarcomas. Refractory hematologic malignancies, endometrial cancer, breast cancer	Ridaforolimus received FDA approval in the year 2011 for sarcoma during NCT00093080 and NCT00538239. NCT00086125, Phase II, Year-2004 NCT00122343, Phase II, Year-2005 NCT00739830, Phase II, Year-2008 NCT00736970, Phase II, Year-2008 NCT01431547, Phase I, Year-2012	Remarkable potential as an anticancer drug in a variety of human malignancies	Adverse effects include mucositis, myelosuppression, thrombocytopenia, rash, and nausea.	Sarcoma cell lines RH30 = 2–5 nM SYO-I = 23.1 ± 4.6 HS-SY-II = 10.9 ± 2.7	^271–277^
Vistusertib (AZD2014)	mTORC1 & mTORC2	ER-positive breast cancer, symptomatic meningioma, platinum-resistance ovarian carcinoma, and lymphoma	NCT02216786, Phase II, Year-2014 NCT02831257, Phase II, Year 2016 NCT03071874, Phase II, Year-2017 isrctn.org, the Identifier number is ISRCTN16426935.	Potential anticancer agent for inducing apoptosis, cell cycle arrest, and autophagy in human malignancies.	The adverse effects include hyperglycemia, Fatigue, lymphopenia, and diarrhea.	IC_50_ concentration varies from 30 to 500 nM for the ER-positive breast cancer cell lines.	^278,279^
AZD8055	mTORC1 & mTORC2	Glioma, advanced solid tumors, leukemia	NCT 01316809, Phase I, Year 2011 NCT00973076, Phase I, Year 2009	Improved selectivity and potency	Adverse effects, including hyperglycemia and thrombocytopenia	≤2 nM for BT474 breast cancer. 20–50 nM for RCC cell lines and 400 nM for benign RCC. Glioblastoma cell lines IC_50_ range from 100 nM to 500 nM.	^280–284^
Sapanisertib (MLN0128, TAK-228, INK128,)	mTORC1 & mTORC2	mCRPC, Metastatic thyroid cancer, endometrial, RCC, and advanced solid tumors. Bladder cancer, ER^+^ breast cancer, & soft-tissue sarcoma	NCT02091531, Phase II, Year-2014 NCT02244463, Phase II, Year-2015 NCT01058707, Phase I, Year-2010 NCT02412722, Phase I, Year-2015 NCT03047213, Phase II, Year 2016 NCT02988986, Phase II, Year-2017 NCT02987959, Phase II, Year-2017	Greater potency with enhanced anticancer efficacy, selectivity, and safety.	Adverse effects, including hyperglycemia and hematologic toxicity	IC_50_ value varies from 20 to 100 nM in prostate cancer cells (LNCaP, and DU145); 1–162 nM in breast cancer cell lines; 2–150 nM in different sarcoma cells including RH30, RMS559, A673, TC32, LPS141, SAOS2, ST8814. BC-1 16.20 ± 4.94 nM; BCBL-137.43 ± 13.13 nM; BC-3, 47.56 ± 7.48, BCP-19.71 ± 0.76 nM.	^297–310^
Onatasertib (CC-223)	mTORC1 & mTORC2	GBM, HCC, NSCLC, Multiple myeloma, DLBCL, HR^+^ breast cancer, neuroendocrine non-pancreatic origin tumors	NCT01177397, Phase I/II, Year-2010 NCT03591965 and NCT02031419 are active.	ATP-competitive inhibitor, durable response, high selectivity, and efficacy.	The side effects include nausea, vomiting, headache, diarrhea, stomatitis, hyperglycemia, decreased appetite, and asthenia.	0.2–0.35 μM in H929 (B-lymphocyte). 88.17 ± 6.32 nM and 64.32 ± 5.21 nM in the CaOV3 and SKOV3 (ovarian carcinoma cell lines), respectively.	^311–314^
OSI-027	mTORC1 & mTORC2	Advanced solid tumors, multiple myeloma, lymphoma, and pancreatic cancer	NCT00698243, Phase I, Year-2008	Enhanced the inhibition of the mTOR pathway leading to promising anticancer activity	Adverse effects, including hyperglycemia and neutropenia	22 nM and 500 nM in MM.1 S and JJN3 (multiple myeloma), respectively. 1–30 μM in BxPC3, CFPAC1, PANC1, pancreatic cell lines	^315–320^
Dual PI3K/mTOR inhibitors							
Dactolisib (BEZ235)	PI3K & mTOR	PNET, breast cancer	NCT01658436, Phase II, Year-2012 NCT00620594, Phase I/II, Year-2006	Modest clinical efficacy and enhanced efficacy of the immune checkpoint inhibitors	Fatigue, diarrhea, nausea, and mucositis	Ranging from 100 to 2000 nM in breast cancer cell lines (MCF-7, MDAMB231). Ranging from 50 to 300 nM in myeloma cell lines (U266, KM3, RPMI8226).	^322–327^
Paxalisib (GDC-0084)	PI3K & mTOR	GBM	NCT03522298, Phase II, Year-2018 NCT05009992, Phase II, Year-2021 NCT01547546, Phase I, Year-2012	FDA approved for GBM with superior anticancer efficacy.	Hyperglycemia, rashes, mucositis	IC_50_ ranged from 300 nM to 1100 nM in glioma cell lines	^331–334^
Apitolisib (GDC-0980)	PI3K & mTOR	PC	NCT01485861, Phase II, Year-2019 NCT01437566, Phase II, Year 2011 NCT01332604, Phase I, Year 2011		Fatigue, diarrhea, rash, nausea, loss of appetite, Mucosal inflammation, hyperglycemia, vomiting, pruritus, liver dysfunction	The IC_50_ value for prostate, breast, and NSCLC < 200. The pancreatic carcinoma and melanoma ranged from 200 to 500 nmol/L. GDC-0980 was reported with <500 nmol/L in 124 out of the 167 cell lines tested.	^335–337^
Voxtalisib (XL765 or SAR245409)	PI3K & mTOR	Lymphoma, ovarian cancer, GBM	NCT01403636, Phase II, Year 2011 NCT01936363, Phase II, Year-2013 NCT01596270, Phase I, Year-2012	Promising anticancer efficacy	Fatigue, diarrhea, nausea, pyrexia, decreased appetite, and cough		^338–341^
Bimiralisib (PQR309)	PI3K & mTOR	Refractory lymphoma, primary CNS lymphoma, breast cancer	NCT02249429, Phase II, Year-2015 NCT02669511, Phase II, Year 2016		The side effects of the compound were sweating at night, nausea, crankiness, memory problems	174–324 nmol/L in lymphoma cell lines	^279,329,342,343^
Samotolisib (LY3023414)	PI3K & mTOR	mCRPC and other advanced cancers	NCT02407054, Phase II, Year-2015 NCT01655225, Phase I, Year 2012	Samotolisib was noticed with high bioavailability and selectively inhibits PI3K as well as mTOR kinase. Highly effective in clinical trials for human cancers.	Samotolisib was associated with nausea, vomiting sensation, loss of appetite, diarrhea, and headache.	The following cholangiocarcinoma cell lines have IC_50_ in μM. HuCCT1 = 0.52, OZ = 0.45 NCC-BD1 = 0.41, NCC-BD2 = 0.16, NCC-BD3 = 0.32, NCC-CC6–1 = 0.71, NCC-CC4-1 = 0.23	^344,345^
Gedatolisib (PF-05212384, PKI-587)	PI3K, kinase site of mTOR	TNBC, BRCA1/2-positive, HER2^−^ breast and endometrial cancers. Advanced squamous cell Lung, Pancreatic, Head & Neck, and Other Solid Tumors	NCT03911973, Phase II, Year-2019 NCT03698383, Phase II, Year-2018 NCT05501886, Phase III, Year-2022 NCT01420081, Year 2011 NCT01347866, Phase I, Year 2011 NCT00940498, Phase I, Year 2009 NCT03065062, Phase I, Year-2023	Gedatolisib suppresses the tumor burden in patients with advanced solid tumors. Gedatolisib in combination with chemotherapies displayed greater clinical benefit in relapse or refractory cancer patients	Gedatolisib was found to cause neutropenia, abnormal liver function profiles, thrombocytopenia, and anemia.	IC_50_ value was 340 nM in SW620 and 7.09 μM in adriamycin resistance SW620 cells. IC_50_ value was 0.41 μM, and 3.9 μM in LS180 and LS180/MX cells, respectively. The following cell lines have IC_50_ in μmol/L MIAPaCa = 0.022 T47D = 0.002 MCF = 0.036 HT29 = 0.037 HTB44 = 0.43 PC3 = 0.011 A549 = 0.02 H460 = 0.05 DLD1 = 0.31 U87 = 0.012 KB = 0.005	^348–357^

*ALL* acute lymphoblastic leukemia, *AMPECT* advanced malignant PEComa, *CNS* central nervous system, *DLBCL* diffuse large B-cell lymphoma, *ER* *+* estrogen receptor-positive, *FDA* Food and Drug Administration, *FKBP12* FK506-binding protein-12, *GBM* glioblastoma, *HCC* hepatocellular carcinoma, *HER2* human epidermal growth factor receptor 2, *HNSCC* head and neck squamous cell carcinoma, *HR* *+* hormone receptor-positive, *IC*_*50*_ half-maximal inhibitory concentration, *ISRCTN* international standard randomized controlled trial number, *mCRPC* metastatic castration-resistant prostate cancer, *mTOR* mammalian target of rapamycin, *mTORC1* mTOR complex 1, *mTORC2* mTOR complex 2, *NCT* national clinical trial, *NSCLC* non-small cell lung carcinoma, *ORR* overall response rate, *OS* osteosarcoma, *PC* prostate cancer, *PEComa* perivascular epithelioid cell tumor, *PI3K* phosphoinositide 3-kinase, *PNET* pancreatic neuroendocrine tumor, *RCC* renal cell carcinoma, *SEGA* subependymal giant-cell astrocytoma, *TNBC* triple-negative breast cancer, *TSC1* target tuberous sclerosis 1, *TSC2* target tuberous sclerosis 2

## Conclusion and future perspectives

The discovery of mTOR was one of the breakthroughs in understanding the regulation associated with cellular growth, remodeling of the cytoskeleton, cellular metabolism, immune responses, and autophagy in human physiology and cellular homeostasis. The mTOR signaling was noticed to modulate multiple signaling networks to integrate growth factors, amino acids, nutrients, sterols, and nucleotides. The emerging pieces of evidence have displayed that mTOR activation plays a crucial role in aging, age-related neurological disorders, diabetes, and human malignancies. This has been noticed that inhibition of the mTORC1 signaling prolonged life expectancy while boosting immunity which helps in preventing the onset of age-related, neurological disorders, cancer, and several metabolic diseases in both mammals and humans. Given the fact that mTORC1 is involved in the aging processes, immunity, and other key physiological activities, mTORC1-specific inhibitors can be developed that can produce desirable results in clinical trials. Also, the duration of the mTOR inhibitors should be optimized to prevent side effects like immunosuppression and glucose intolerance. During the last decade, extensive research studies have revealed that the mTOR pathway is highly dysregulated in cancers due to hyperactivation of oncogenic signaling cascades, hotspot mutations/amplifications in the oncogenes, and the deletion or loss of function mutations in tumor suppressor genes. Also, mTOR signaling cascade is well known to influence gene transcription and translation to control cellular proliferation, cytoskeleton, cellular migration, differentiation, tumor metabolism, and immune responses in the tumor microenvironment. Hence, targeting the mTOR pathway provided an opportunity for the discovery of novel therapeutics against human cancers and other diseases. In this direction, several efforts have been taken which led to the discovery of the first-generation mTOR inhibitors called Rapalogs. However, clinical efficacy of the Rapalogs was correlated with a modest anticancer effect in multiple trials on cancer patients. Several mTORC1 inhibitors which include sirolimus, everolimus, temsirolimus, and ridaforolimus were approved through FDA for the management and treatment of several types of human malignancies in clinics. Hence, several groups and pharma companies have focused on the development of new combination therapeutic approaches along with the designing, synthesis, and validation of dual inhibitors (PI3K/mTOR) for effective and relapse-free therapy to overcome resistance. Furthermore, the dual inhibitors targeting PI3K/mTOR axis, inhibition of parallel pathways, and targeted/non-targeted drug combinations were tested in preclinical and clinical studies to figure out the best possibility for mTOR inhibition. The results of these combinations were promising. Currently, the development of third-generation mTOR inhibitors (Rapalink-1) displayed promising results in clinical trials because of their ability to inhibit the mutant kinase activity. During the past decade, the libraries of potential mTOR inhibitors included a variety of scaffolds like urea, quinolines, pyridines, and pyridopyrimidines. Apart from these various small molecules have been designed, manufactured, and assessed as mTOR inhibitors with promising anticancer efficacy. In the future, we hope that the Identification of novel and specific inhibitors of the mTOR pathway or mTOR-associated pathways that can eradicate cancer cells, CSCs, drug resistance, and autophagy either alone or in combination with chemotherapeutic drugs and immunotherapeutic agents could be of great importance for the treatment of human malignancies and research purposes. Recently, Nab-sirolimus (the albumin-bound nanoparticle of sirolimus) has been approved by the FDA for clinical use against human cancer. Therefore, the nano-formulation of the mTOR signaling pathway inhibitors should be a priority for better efficacy and limited toxicity. Moreover, preclinical and clinical studies are required to assess the safety, efficacy, and molecular mechanism of the other chemotherapeutic drugs, and immune therapies in combination with mTOR inhibitors. This could lead to the invention of more effective and less toxic treatment regimens that can improve the relapse-free survival of cancer patients.

## References

[1] Saxton RA, Sabatini DM (2017). mTOR signaling in growth, metabolism, and disease. Cell.

[2] Laplante M, Sabatini DM (2012). mTOR signaling in growth control and disease. Cell.

[3] Yin Y (2016). mTORC2 promotes type I insulin-like growth factor receptor and insulin receptor activation through the tyrosine kinase activity of mTOR. Cell Res..

[4] Sehgal SN, Baker H, Vezina C (1975). Rapamycin (AY-22,989), a new antifungal antibiotic. II. Fermentation, isolation and characterization. J. Antibiot..

[5] Zou Z, Tao T, Li H, Zhu X (2020). mTOR signaling pathway and mTOR inhibitors in cancer: progress and challenges. Cell Biosci..

[6] Loewith R (2002). Two TOR complexes, only one of which is rapamycin sensitive, have distinct roles in cell growth control. Mol. Cell.

[7] Popova, N. V. & Jucker, M. The role of mTOR signaling as a therapeutic target in cancer. *Int. J. Mol. Sci*. **22**, 1743 (2021). 10.3390/ijms22041743 PMC7916160 33572326

[8] Hara K (2002). Raptor, a binding partner of target of rapamycin (TOR), mediates TOR action. Cell.

[9] Hua H (2019). Targeting mTOR for cancer therapy. J. Hematol. Oncol..

[10] El-Tanani M (2023). Role of mammalian target of rapamycin (mTOR) signalling in oncogenesis. Life Sci..

[11] Kim DH (2003). GbetaL, a positive regulator of the rapamycin-sensitive pathway required for the nutrient-sensitive interaction between raptor and mTOR. Mol. Cell.

[12] Yang Q, Inoki K, Ikenoue T, Guan KL (2006). Identification of Sin1 as an essential TORC2 component required for complex formation and kinase activity. Genes Dev..

[13] Sarbassov DD (2004). Rictor, a novel binding partner of mTOR, defines a rapamycin-insensitive and raptor-independent pathway that regulates the cytoskeleton. Curr. Biol..

[14] Pearce LR (2007). Identification of Protor as a novel Rictor-binding component of mTOR complex-2. Biochem. J..

[15] You M (2023). Signaling pathways in cancer metabolism: mechanisms and therapeutic targets. Signal Transduct. Target Ther..

[16] Dowling RJ, Topisirovic I, Fonseca BD, Sonenberg N (2010). Dissecting the role of mTOR: lessons from mTOR inhibitors. Biochim. Biophys. Acta.

[17] Rabanal-Ruiz Y, Otten EG, Korolchuk VI (2017). mTORC1 as the main gateway to autophagy. Essays Biochem..

[18] Benjamin D, Colombi M, Moroni C, Hall MN (2011). Rapamycin passes the torch: a new generation of mTOR inhibitors. Nat. Rev. Drug Discov..

[19] Hanahan D, Weinberg RA (2011). Hallmarks of cancer: the next generation. Cell.

[20] Garg M, Braunstein G, Koeffler HP (2014). LAMC2 as a therapeutic target for cancers. Expert Opin. Ther. Targets.

[21] Garg M (2015). Establishment and characterization of novel human primary and metastatic anaplastic thyroid cancer cell lines and their genomic evolution over a year as a prima graft. J. Clin. Endocrinol. Metab..

[22] Populo H, Lopes JM, Soares P (2012). The mTOR signalling pathway in human cancer. Int. J. Mol. Sci..

[23] Kirtonia A (2022). Overexpression of laminin-5 gamma-2 promotes tumorigenesis of pancreatic ductal adenocarcinoma through EGFR/ERK1/2/AKT/mTOR cascade. Cell Mol. Life Sci..

[24] Nepstad I (2019). Effects of insulin and pathway inhibitors on the PI3K-Akt-mTOR phosphorylation profile in acute myeloid leukemia cells. Signal Transduct. Target Ther..

[25] He Y (2022). Targeting signaling pathways in prostate cancer: mechanisms and clinical trials. Signal Transduct. Target Ther..

[26] Murugan AK (2019). mTOR: Role in cancer, metastasis and drug resistance. Semin Cancer Biol..

[27] Garber K (2009). Targeting mTOR: something old, something new. J. Natl Cancer Inst..

[28] Yang H (2016). 4.4 A resolution Cryo-EM structure of human mTOR complex 1. Protein Cell.

[29] Yang H (2013). mTOR kinase structure, mechanism and regulation. Nature.

[30] Unni N, Arteaga CL (2019). Is dual mTORC1 and mTORC2 therapeutic blockade clinically feasible in cancer?. JAMA Oncol..

[31] Yip CK (2010). Structure of the human mTOR complex I and its implications for rapamycin inhibition. Mol. Cell.

[32] Yuan HX, Guan KL (2016). Structural insights of mTOR complex 1. Cell Res..

[33] Baretic D (2016). Tor forms a dimer through an N-terminal helical solenoid with a complex topology. Nat. Commun..

[34] Guertin DA, Sabatini DM (2007). Defining the role of mTOR in cancer. Cancer Cell.

[35] Scaiola, A. et al. The 3.2-A resolution structure of human mTORC2. *Sci Adv*. **6**, eabc1251 (2020). 10.1126/sciadv.abc1251 PMC7673708 33158864

[36] Pearce LR (2011). Protor-1 is required for efficient mTORC2-mediated activation of SGK1 in the kidney. Biochem. J..

[37] Harwood FC (2018). ETV7 is an essential component of a rapamycin-insensitive mTOR complex in cancer. Sci. Adv..

[38] Laplante M, Sabatini DM (2009). mTOR signaling at a glance. J. Cell Sci..

[39] Menon S (2014). Spatial control of the TSC complex integrates insulin and nutrient regulation of mTORC1 at the lysosome. Cell.

[40] Sengupta S, Peterson TR, Sabatini DM (2010). Regulation of the mTOR complex 1 pathway by nutrients, growth factors, and stress. Mol. Cell.

[41] Gwinn DM (2008). AMPK phosphorylation of raptor mediates a metabolic checkpoint. Mol. Cell.

[42] Dai X (2023). AMPK-dependent phosphorylation of the GATOR2 component WDR24 suppresses glucose-mediated mTORC1 activation. Nat. Metab..

[43] Efeyan A (2013). Regulation of mTORC1 by the Rag GTPases is necessary for neonatal autophagy and survival. Nature.

[44] Kalender A (2010). Metformin, independent of AMPK, inhibits mTORC1 in a rag GTPase-dependent manner. Cell Metab..

[45] Yue S, Li G, He S, Li T (2022). The central role of mTORC1 in amino acid sensing. Cancer Res..

[46] Kim E (2008). Regulation of TORC1 by Rag GTPases in nutrient response. Nat. Cell Biol..

[47] Sancak Y (2008). The Rag GTPases bind raptor and mediate amino acid signaling to mTORC1. Science.

[48] Lama-Sherpa TD, Jeong MH, Jewell JL (2023). Regulation of mTORC1 by the Rag GTPases. Biochem. Soc. Trans..

[49] Bar-Peled L, Schweitzer LD, Zoncu R, Sabatini DM (2012). Ragulator is a GEF for the rag GTPases that signal amino acid levels to mTORC1. Cell.

[50] Cui Z, Joiner AMN, Jansen RM, Hurley JH (2023). Amino acid sensing and lysosomal signaling complexes. Curr. Opin. Struct. Biol..

[51] Jung J, Genau HM, Behrends C (2015). Amino acid-dependent mTORC1 regulation by the lysosomal membrane protein SLC38A9. Mol. Cell Biol..

[52] Wolfson RL (2017). KICSTOR recruits GATOR1 to the lysosome and is necessary for nutrients to regulate mTORC1. Nature.

[53] Bar-Peled L (2013). A Tumor suppressor complex with GAP activity for the Rag GTPases that signal amino acid sufficiency to mTORC1. Science.

[54] Parmigiani A (2014). Sestrins inhibit mTORC1 kinase activation through the GATOR complex. Cell Rep..

[55] Ye J (2015). GCN2 sustains mTORC1 suppression upon amino acid deprivation by inducing Sestrin2. Genes Dev..

[56] Duvel K (2010). Activation of a metabolic gene regulatory network downstream of mTOR complex 1. Mol. Cell.

[57] Saxton RA (2016). Structural basis for leucine sensing by the Sestrin2-mTORC1 pathway. Science.

[58] Petit CS, Roczniak-Ferguson A, Ferguson SM (2013). Recruitment of folliculin to lysosomes supports the amino acid-dependent activation of Rag GTPases. J. Cell Biol..

[59] Jewell JL (2015). Differential regulation of mTORC1 by leucine and glutamine. Science.

[60] Matsumoto A (2017). mTORC1 and muscle regeneration are regulated by the LINC00961-encoded SPAR polypeptide. Nature.

[61] Yan G (2023). Genome-wide CRISPR screens identify ILF3 as a mediator of mTORC1-dependent amino acid sensing. Nat. Cell Biol..

[62] Jiang C (2023). Ring domains are essential for GATOR2-dependent mTORC1 activation. Mol. Cell.

[63] Liu P (2015). PtdIns(3,4,5)P3-dependent activation of the mTORC2 kinase complex. Cancer Discov..

[64] Yang G, Murashige DS, Humphrey SJ, James DE (2015). A positive feedback loop between Akt and mTORC2 via SIN1 phosphorylation. Cell Rep..

[65] Harrington LS (2004). The TSC1-2 tumor suppressor controls insulin-PI3K signaling via regulation of IRS proteins. J. Cell Biol..

[66] Hsu PP (2011). The mTOR-regulated phosphoproteome reveals a mechanism of mTORC1-mediated inhibition of growth factor signaling. Science.

[67] Yu Y (2011). Phosphoproteomic analysis identifies Grb10 as an mTORC1 substrate that negatively regulates insulin signaling. Science.

[68] Lacher MD, Pincheira RJ, Castro AF (2011). Consequences of interrupted Rheb-to-AMPK feedback signaling in tuberous sclerosis complex and cancer. Small GTPases.

[69] Fan H (2021). Critical role of mTOR in regulating aerobic glycolysis in carcinogenesis (review). Int. J. Oncol..

[70] Yecies JL, Manning BD (2011). Transcriptional control of cellular metabolism by mTOR signaling. Cancer Res..

[71] Magaway, C., Kim, E. & Jacinto, E. Targeting mTOR and metabolism in cancer: lessons and innovations. *Cells*. **8**, 1584 (2019). 10.3390/cells8121584 PMC6952948 31817676

[72] Sengupta S, Sahasrabuddhe D, Wangikar PP (2022). Transporter engineering for the development of cyanobacteria as cell factories: a text analytics guided survey. Biotechnol. Adv..

[73] Peterson TR (2011). mTOR complex 1 regulates lipin 1 localization to control the SREBP pathway. Cell.

[74] Ben-Sahra I, Howell JJ, Asara JM, Manning BD (2013). Stimulation of de novo pyrimidine synthesis by growth signaling through mTOR and S6K1. Science.

[75] Robitaille AM (2013). Quantitative phosphoproteomics reveal mTORC1 activates de novo pyrimidine synthesis. Science.

[76] Holz MK, Ballif BA, Gygi SP, Blenis J (2021). mTOR and S6K1 mediate assembly of the translation preinitiation complex through dynamic protein interchange and ordered phosphorylation events. Cell.

[77] Dorrello NV (2006). S6K1- and betaTRCP-mediated degradation of PDCD4 promotes protein translation and cell growth. Science.

[78] Ma XM (2008). SKAR links pre-mRNA splicing to mTOR/S6K1-mediated enhanced translation efficiency of spliced mRNAs. Cell.

[79] Hsieh AC (2012). The translational landscape of mTOR signalling steers cancer initiation and metastasis. Nature.

[80] Thoreen CC (2009). An ATP-competitive mammalian target of rapamycin inhibitor reveals rapamycin-resistant functions of mTORC1. J. Biol. Chem..

[81] Thoreen CC (2012). A unifying model for mTORC1-mediated regulation of mRNA translation. Nature.

[82] Braun C (2021). p38 regulates the tumor suppressor PDCD4 via the TSC-mTORC1 pathway. Cell Stress.

[83] Brunn GJ (1997). Phosphorylation of the translational repressor PHAS-I by the mammalian target of rapamycin. Science.

[84] Tian, T., Li, X. & Zhang, J. mTOR signaling in cancer and mTOR inhibitors in solid tumor targeting therapy. *Int. J. Mol Sci*. **20**, 755 (2019). 10.3390/ijms20030755 PMC6387042 30754640

[85] Conciatori, F. et al. mTOR cross-talk in cancer and potential for combination therapy. *Cancers* **10**, 23 (2018). 10.3390/cancers10010023 PMC5789373 29351204

[86] Grabiner BC (2014). A diverse array of cancer-associated MTOR mutations are hyperactivating and can predict rapamycin sensitivity. Cancer Discov..

[87] Cheng H (2015). RICTOR amplification defines a novel subset of patients with lung cancer who may benefit from treatment with mTORC1/2 inhibitors. Cancer Discov..

[88] Morrison Joly M (2016). Rictor/mTORC2 drives progression and therapeutic resistance of HER2-amplified breast cancers. Cancer Res..

[89] El Shamieh S (2018). RICTOR gene amplification is correlated with metastasis and therapeutic resistance in triple-negative breast cancer. Pharmacogenomics.

[90] Berarducci JP, Joseph RW (1994). A bone collection suction device for use during endosseous implant placement. J. Oral. Maxillofac. Surg..

[91] Zhang Y (2017). A pan-cancer proteogenomic atlas of PI3K/AKT/mTOR pathway alterations. Cancer Cell.

[92] Gao Y (2016). Rheb1 promotes tumor progression through mTORC1 in MLL-AF9-initiated murine acute myeloid leukemia. J. Hematol. Oncol..

[93] Ghosh AP (2015). Point mutations of the mTOR-RHEB pathway in renal cell carcinoma. Oncotarget.

[94] Guertin DA (2009). mTOR complex 2 is required for the development of prostate cancer induced by Pten loss in mice. Cancer Cell.

[95] Sengupta S (2022). Transition of amyloid/mutant p53 from tumor suppressor to an oncogene and therapeutic approaches to ameliorate metastasis and cancer stemness. Cancer Cell Int..

[96] DeGraffenried LA (2004). Reduced PTEN expression in breast cancer cells confers susceptibility to inhibitors of the PI3 kinase/Akt pathway. Ann. Oncol..

[97] Shi Y (2002). Enhanced sensitivity of multiple myeloma cells containing PTEN mutations to CCI-779. Cancer Res..

[98] Milam MR (2007). Reduced progression of endometrial hyperplasia with oral mTOR inhibition in the Pten heterozygote murine model. Am. J. Obstet. Gynecol..

[99] Jiao Y (2011). DAXX/ATRX, MEN1, and mTOR pathway genes are frequently altered in pancreatic neuroendocrine tumors. Science.

[100] Sjodahl G (2011). A systematic study of gene mutations in urothelial carcinoma; inactivating mutations in TSC2 and PIK3R1. PLoS ONE.

[101] Platt FM (2009). Spectrum of phosphatidylinositol 3-kinase pathway gene alterations in bladder cancer. Clin. Cancer Res..

[102] Shabna A (2023). Long non-coding RNAs: Fundamental regulators and emerging targets of cancer stem cells. Biochim. Biophys. Acta Rev. Cancer.

[103] Sharma A (2021). Long non-coding RNAs orchestrate various molecular and cellular processes by modulating epithelial-mesenchymal transition in head and neck squamous cell carcinoma. Biochim. Biophys. Acta Mol. Basis Dis..

[104] Garg M, Sethi G (2021). Emerging role of long non-coding RNA (lncRNA) in human malignancies: a unique opportunity for precision medicine. Cancer Lett..

[105] Chen B (2022). Targeting non-coding RNAs to overcome cancer therapy resistance. Signal Transduct. Target Ther..

[106] Kanojia D (2022). Transcriptome analysis identifies TODL as a novel lncRNA associated with proliferation, differentiation, and tumorigenesis in liposarcoma through FOXM1. Pharm. Res..

[107] Yadav B (2021). LncRNAs associated with glioblastoma: from transcriptional noise to novel regulators with a promising role in therapeutics. Mol. Ther. Nucleic Acids.

[108] Pandya G (2020). The implication of long non-coding RNAs in the diagnosis, pathogenesis and drug resistance of pancreatic ductal adenocarcinoma and their possible therapeutic potential. Biochim. Biophys. Acta Rev. Cancer.

[109] Kirtonia A (2022). Long noncoding RNAs: a novel insight in the leukemogenesis and drug resistance in acute myeloid leukemia. J. Cell Physiol..

[110] Huang T (2018). Long noncoding RNAs in the mTOR signaling network: biomarkers and therapeutic targets. Apoptosis.

[111] Aboudehen K (2020). Regulation of mTOR signaling by long non-coding RNA. Biochim Biophys. Acta Gene Regul. Mech..

[112] Du Y (2018). lncRNA DLEU1 contributes to tumorigenesis and development of endometrial carcinoma by targeting mTOR. Mol. Carcinog..

[113] Chen JF (2018). STAT3-induced lncRNA HAGLROS overexpression contributes to the malignant progression of gastric cancer cells via mTOR signal-mediated inhibition of autophagy. Mol. Cancer.

[114] Liu X (2016). LncRNA NBR2 engages a metabolic checkpoint by regulating AMPK under energy stress. Nat. Cell Biol..

[115] Malakar P (2017). Long noncoding RNA MALAT1 promotes hepatocellular carcinoma development by SRSF1 upregulation and mTOR activation. Cancer Res..

[116] Ji J (2015). LINC00152 promotes proliferation in hepatocellular carcinoma by targeting EpCAM via the mTOR signaling pathway. Oncotarget.

[117] Wu ZR (2018). Inhibition of mTORC1 by lncRNA H19 via disrupting 4E-BP1/Raptor interaction in pituitary tumours. Nat. Commun..

[118] Shermane Lim YW (2021). The double-edged sword of H19 lncRNA: Insights into cancer therapy. Cancer Lett..

[119] Yu T (2017). MetaLnc9 facilitates lung cancer metastasis via a PGK1-activated AKT/mTOR pathway. Cancer Res..

[120] Dong, S., Zhang, X. & Liu, D. Overexpression of long noncoding RNA GAS5 suppresses tumorigenesis and development of gastric cancer by sponging miR-106a-5p through the Akt/mTOR pathway. *Biol Open*. **8**, bio041343 (2019). 10.1242/bio.041343 PMC6602335 31182630

[121] Huo JF, Chen XB (2019). Long noncoding RNA growth arrest-specific 5 facilitates glioma cell sensitivity to cisplatin by suppressing excessive autophagy in an mTOR-dependent manner. J. Cell Biochem..

[122] Yang Y, Chen D, Liu H, Yang K (2019). Increased expression of lncRNA CASC9 promotes tumor progression by suppressing autophagy-mediated cell apoptosis via the AKT/mTOR pathway in oral squamous cell carcinoma. Cell Death Dis..

[123] Chen W (2023). LncTUG1 contributes to the progression of hepatocellular carcinoma via the miR-144-3p/RRAGD axis and mTOR/S6K pathway. Sci. Rep..

[124] Wang Y (2015). CRNDE, a long-noncoding RNA, promotes glioma cell growth and invasion through mTOR signaling. Cancer Lett..

[125] Zhu Y (2016). HULC long noncoding RNA silencing suppresses angiogenesis by regulating ESM-1 via the PI3K/Akt/mTOR signaling pathway in human gliomas. Oncotarget.

[126] Li Z (2014). Long non-coding RNA UCA1 promotes glycolysis by upregulating hexokinase 2 through the mTOR-STAT3/microRNA143 pathway. Cancer Sci..

[127] Li P (2020). ZNNT1 long noncoding RNA induces autophagy to inhibit tumorigenesis of uveal melanoma by regulating key autophagy gene expression. Autophagy.

[128] Yang L (2020). Targeting cancer stem cell pathways for cancer therapy. Signal Transduct. Target Ther..

[129] Sneha S (2020). The hedgehog pathway regulates cancer stem cells in serous adenocarcinoma of the ovary. Cell Oncol..

[130] Bindhya S (2021). Development and in vitro characterisation of an induced pluripotent stem cell model of ovarian cancer. Int. J. Biochem Cell Biol..

[131] Zhou J (2007). Activation of the PTEN/mTOR/STAT3 pathway in breast cancer stem-like cells is required for viability and maintenance. Proc. Natl. Acad. Sci. USA.

[132] Nishitani S, Horie M, Ishizaki S, Yano H (2013). Branched chain amino acid suppresses hepatocellular cancer stem cells through the activation of mammalian target of rapamycin. PLoS ONE.

[133] Eckerdt FD (2020). Combined PI3Kalpha-mTOR targeting of glioma stem cells. Sci. Rep..

[134] Sunayama J (2010). Dual blocking of mTor and PI3K elicits a prodifferentiation effect on glioblastoma stem-like cells. Neuro Oncol..

[135] Corominas-Faja B (2013). Nuclear reprogramming of luminal-like breast cancer cells generates Sox2-overexpressing cancer stem-like cellular states harboring transcriptional activation of the mTOR pathway. Cell Cycle.

[136] Dubrovska A (2009). The role of PTEN/Akt/PI3K signaling in the maintenance and viability of prostate cancer stem-like cell populations. Proc. Natl. Acad. Sci. USA.

[137] Jung MJ (2013). Upregulation of CXCR4 is functionally crucial for maintenance of stemness in drug-resistant non-small cell lung cancer cells. Oncogene.

[138] Chang WW (2013). The expression and significance of insulin-like growth factor-1 receptor and its pathway on breast cancer stem/progenitors. Breast Cancer Res..

[139] Hoshii T (2012). mTORC1 is essential for leukemia propagation but not stem cell self-renewal. J. Clin. Investig..

[140] Airiau K (2013). PI3K/mTOR pathway inhibitors sensitize chronic myeloid leukemia stem cells to nilotinib and restore the response of progenitors to nilotinib in the presence of stem cell factor. Cell Death Dis..

[141] Matsumoto K (2009). mTOR signal and hypoxia-inducible factor-1 alpha regulate CD133 expression in cancer cells. Cancer Res..

[142] Yang Z (2011). Transient mTOR inhibition facilitates continuous growth of liver tumors by modulating the maintenance of CD133+ cell populations. PLoS ONE.

[143] Lin F (2017). PI3K-mTOR pathway inhibition exhibits efficacy against high-grade glioma in clinically relevant mouse models. Clin. Cancer Res..

[144] Hurvitz SA (2015). In vitro activity of the mTOR inhibitor everolimus, in a large panel of breast cancer cell lines and analysis for predictors of response. Breast Cancer Res. Treat..

[145] Park HS (2014). Synergistic antitumor effect of NVP-BEZ235 and sunitinib on docetaxel-resistant human castration-resistant prostate cancer cells. Anticancer Res..

[146] Wu CP (2017). Overexpression of ATP-binding cassette subfamily G member 2 confers resistance to phosphatidylinositol 3-kinase inhibitor PF-4989216 in cancer cells. Mol. Pharm..

[147] Wu CP (2020). Overexpression of ABCB1 and ABCG2 contributes to reduced efficacy of the PI3K/mTOR inhibitor samotolisib (LY3023414) in cancer cell lines. Biochem. Pharm..

[148] Rodrik-Outmezguine VS (2016). Overcoming mTOR resistance mutations with a new-generation mTOR inhibitor. Nature.

[149] Wagle N (2014). Response and acquired resistance to everolimus in anaplastic thyroid cancer. N. Engl. J. Med..

[150] Mizushima N, Levine B, Cuervo AM, Klionsky DJ (2008). Autophagy fights disease through cellular self-digestion. Nature.

[151] Levine B, Klionsky DJ (2017). Autophagy wins the 2016 Nobel Prize in Physiology or Medicine: breakthroughs in baker’s yeast fuel advances in biomedical research. Proc. Natl Acad. Sci. USA.

[152] Deleyto-Seldas N, Efeyan A (2021). The mTOR-autophagy axis and the control of metabolism. Front. Cell Dev. Biol..

[153] Clark SL (1957). Cellular differentiation in the kidneys of newborn mice studies with the electron microscope. J. Biophys. Biochem. Cytol..

[154] Meijer AJ, Lorin S, Blommaart EF, Codogno P (2015). Regulation of autophagy by amino acids and MTOR-dependent signal transduction. Amino Acids.

[155] Kim YC, Guan KL (2015). mTOR: a pharmacologic target for autophagy regulation. J. Clin. Investig..

[156] Ganley IG (2009). ULK1.ATG13.FIP200 complex mediates mTOR signaling and is essential for autophagy. J. Biol. Chem..

[157] Nwadike, C. et al. AMPK inhibits ULK1-dependent autophagosome formation and lysosomal acidification via distinct mechanisms. *Mol. Cell Biol*. **38**, e00023-18 (2018). 10.1128/MCB.00023-18 PMC5954193 29507183

[158] Nazarko VY, Zhong Q (2013). ULK1 targets Beclin-1 in autophagy. Nat. Cell Biol..

[159] Russell RC (2013). ULK1 induces autophagy by phosphorylating Beclin-1 and activating VPS34 lipid kinase. Nat. Cell Biol..

[160] Itakura E, Kishi C, Inoue K, Mizushima N (2008). Beclin 1 forms two distinct phosphatidylinositol 3-kinase complexes with mammalian Atg14 and UVRAG. Mol. Biol. Cell.

[161] Jung CH (2010). mTOR regulation of autophagy. FEBS Lett..

[162] Luo T (2016). PSMD10/gankyrin induces autophagy to promote tumor progression through cytoplasmic interaction with ATG7 and nuclear transactivation of ATG7 expression. Autophagy.

[163] Liu M (2018). Cytoplasmic liver kinase B1 promotes the growth of human lung adenocarcinoma by enhancing autophagy. Cancer Sci..

[164] Vera-Ramirez L (2018). Autophagy promotes the survival of dormant breast cancer cells and metastatic tumour recurrence. Nat. Commun..

[165] Kirtonia A (2021). Repurposing of drugs: An attractive pharmacological strategy for cancer therapeutics. Semin. Cancer Biol..

[166] Smith AG, Macleod KF (2019). Autophagy, cancer stem cells and drug resistance. J. Pathol..

[167] Vellai T (2003). Genetics: influence of TOR kinase on lifespan in *C. elegans*. Nature.

[168] Jia K, Chen D, Riddle DL (2004). The TOR pathway interacts with the insulin signaling pathway to regulate *C. elegans* larval development, metabolism and life span. Development.

[169] Kapahi P (2004). Regulation of lifespan in Drosophila by modulation of genes in the TOR signaling pathway. Curr. Biol..

[170] Kaeberlein M (2005). Regulation of yeast replicative life span by TOR and Sch9 in response to nutrients. Science.

[171] Lamming DW (2012). Rapamycin-induced insulin resistance is mediated by mTORC2 loss and uncoupled from longevity. Science.

[172] Shindyapina AV (2022). Rapamycin treatment during development extends life span and health span of male mice and *Daphnia magna*. Sci. Adv..

[173] Zhang Y (2014). Rapamycin extends life and health in C57BL/6 mice. J. Gerontol. A Biol. Sci. Med. Sci..

[174] Robida-Stubbs S (2012). TOR signaling and rapamycin influence longevity by regulating SKN-1/Nrf and DAF-16/FoxO. Cell Metab..

[175] Hansen M (2007). Lifespan extension by conditions that inhibit translation in *Caenorhabditis elegans*. Aging Cell.

[176] Selman C (2009). Ribosomal protein S6 kinase 1 signaling regulates mammalian life span. Science.

[177] Filer D (2017). RNA polymerase III limits longevity downstream of TORC1. Nature.

[178] Chen C, Liu Y, Liu Y, Zheng P (2009). mTOR regulation and therapeutic rejuvenation of aging hematopoietic stem cells. Sci. Signal.

[179] Liu L (2020). ER-associated degradation preserves hematopoietic stem cell quiescence and self-renewal by restricting mTOR activity. Blood.

[180] Yilmaz OH (2012). mTORC1 in the Paneth cell niche couples intestinal stem-cell function to calorie intake. Nature.

[181] Bao, L. et al. Amino acid transporter SLC7A5 regulates Paneth cell function to affect the intestinal inflammatory response. Preprint at https://www.biorxiv.org/content/10.1101/2023.01.24.524966v1 (2023).

[182] Artoni, F. et al. Loss of foxo rescues stem cell aging in Drosophila germ line. *eLife* **6**, e27842 (2017). 10.7554/eLife.27842 PMC5644957 28925355

[183] Mannick JB (2014). mTOR inhibition improves immune function in the elderly. Sci. Transl. Med..

[184] Mannick, J. B. et al. TORC1 inhibition enhances immune function and reduces infections in the elderly. *Sci. Transl. Med*. **10**, eaaq1564 (2018). 10.1126/scitranslmed.aaq1564 29997249

[185] Arriola Apelo SI (2016). Alternative rapamycin treatment regimens mitigate the impact of rapamycin on glucose homeostasis and the immune system. Aging Cell.

[186] Lipton JO, Sahin M (2014). The neurology of mTOR. Neuron.

[187] Bercury KK (2014). Conditional ablation of raptor or rictor has differential impact on oligodendrocyte differentiation and CNS myelination. J. Neurosci..

[188] Asato MR, Hardan AY (2004). Neuropsychiatric problems in tuberous sclerosis complex. J. Child Neurol..

[189] Cohen R, Genizi J, Korenrich L (2021). Behavioral symptoms may correlate with the load and spatial location of tubers and with radial migration lines in tuberous sclerosis complex. Front. Neurol..

[190] Zeng LH, Xu L, Gutmann DH, Wong M (2008). Rapamycin prevents epilepsy in a mouse model of tuberous sclerosis complex. Ann. Neurol..

[191] Smialek D, Kotulska K, Duda A, Jozwiak S (2023). Effect of mTOR inhibitors in epilepsy treatment in children with tuberous sclerosis complex under 2 years of age. Neurol. Ther..

[192] Li N (2010). mTOR-dependent synapse formation underlies the rapid antidepressant effects of NMDA antagonists. Science.

[193] Spilman P (2010). Inhibition of mTOR by rapamycin abolishes cognitive deficits and reduces amyloid-beta levels in a mouse model of Alzheimer’s disease. PLoS ONE.

[194] Carosi JM, Sargeant TJ (2023). Rapamycin and Alzheimer disease: a hypothesis for the effective use of rapamycin for treatment of neurodegenerative disease. Autophagy.

[195] Bellozi PMQ (2019). NVP-BEZ235 (Dactolisib) has protective effects in a transgenic mouse model of Alzheimer’s disease. Front. Pharm..

[196] Mafi S (2021). mTOR-mediated regulation of immune responses in cancer and tumor microenvironment. Front. Immunol..

[197] Gonzalez H, Hagerling C, Werb Z (2018). Roles of the immune system in cancer: from tumor initiation to metastatic progression. Genes Dev..

[198] Chi H (2012). Regulation and function of mTOR signalling in T cell fate decisions. Nat. Rev. Immunol..

[199] Yang K (2011). The tumor suppressor Tsc1 enforces quiescence of naive T cells to promote immune homeostasis and function. Nat. Immunol..

[200] Wu Q (2011). The tuberous sclerosis complex-mammalian target of rapamycin pathway maintains the quiescence and survival of naive T cells. J. Immunol..

[201] Finlay DK (2009). Phosphoinositide-dependent kinase 1 controls migration and malignant transformation but not cell growth and proliferation in PTEN-null lymphocytes. J. Exp. Med..

[202] Tamas P (2010). LKB1 is essential for the proliferation of T-cell progenitors and mature peripheral T cells. Eur. J. Immunol..

[203] MacIver NJ (2011). The liver kinase B1 is a central regulator of T cell development, activation, and metabolism. J. Immunol..

[204] Saemann MD (2009). The multifunctional role of mTOR in innate immunity: implications for transplant immunity. Am. J. Transpl..

[205] Powell JD, Lerner CG, Schwartz RH (1999). Inhibition of cell cycle progression by rapamycin induces T cell clonal anergy even in the presence of costimulation. J. Immunol..

[206] Kusaba H (2005). Interleukin-12-induced interferon-gamma production by human peripheral blood T cells is regulated by mammalian target of rapamycin (mTOR). J. Biol. Chem..

[207] Araki K, Youngblood B, Ahmed R (2010). The role of mTOR in memory CD8 T-cell differentiation. Immunol. Rev..

[208] Pollizzi KN (2015). mTORC1 and mTORC2 selectively regulate CD8(+) T cell differentiation. J. Clin. Investig..

[209] Zhang S (2011). Constitutive reductions in mTOR alter cell size, immune cell development, and antibody production. Blood.

[210] Iwata TN, Ramirez-Komo JA, Park H, Iritani BM (2017). Control of B lymphocyte development and functions by the mTOR signaling pathways. Cytokine Growth Factor Rev..

[211] Lazorchak AS (2010). Sin1-mTORC2 suppresses rag and il7r gene expression through Akt2 in B cells. Mol. Cell.

[212] Zhang Y (2014). Rictor is required for early B cell development in bone marrow. PLoS ONE.

[213] Donahue AC, Fruman DA (2003). Proliferation and survival of activated B cells requires sustained antigen receptor engagement and phosphoinositide 3-kinase activation. J. Immunol..

[214] Iwata TN (2016). Conditional disruption of raptor reveals an essential role for mTORC1 in B cell development, survival, and metabolism. J. Immunol..

[215] Gaudette BT (2020). mTORC1 coordinates an immediate unfolded protein response-related transcriptome in activated B cells preceding antibody secretion. Nat. Commun..

[216] Nazari N (2021). The emerging role of microRNA in regulating the mTOR signaling pathway in immune and inflammatory responses. Immunol. Cell Biol..

[217] He Z (2019). Metabolic regulation of dendritic cell differentiation. Front. Immunol..

[218] Warrier, V. U. et al. Engineering anti-cancer nanovaccine based on antigen cross-presentation. *Biosci. Rep*. **39**, BSR20193220 (2019). 10.1042/BSR20193220 PMC6822533 31652460

[219] Gupta B (2022). Plant lectins and their usage in preparing targeted nanovaccines for cancer immunotherapy. Semin. Cancer Biol..

[220] Chiossone, L. & Vivier, E. Bringing natural killer cells to the clinic. *J. Exp. Med*. **219**, e20220830 (2022). 10.1084/jem.20220830 PMC9448638 36066456

[221] Yang, C. et al. mTORC1 and mTORC2 differentially promote natural killer cell development. *eLife* **7**, e35619 (2018). 10.7554/eLife.35619 PMC5976438 29809146

[222] Wang F (2018). Crosstalks between mTORC1 and mTORC2 variagate cytokine signaling to control NK maturation and effector function. Nat. Commun..

[223] Sukhbaatar N, Hengstschlager M, Weichhart T (2016). mTOR-mediated regulation of dendritic cell differentiation and function. Trends Immunol..

[224] Amiel E (2012). Inhibition of mechanistic target of rapamycin promotes dendritic cell activation and enhances therapeutic autologous vaccination in mice. J. Immunol..

[225] Nadella V (2020). Emerging neo adjuvants for harnessing therapeutic potential of M1 tumor associated macrophages (TAM) against solid tumors: enusage of plasticity. Ann. Transl. Med..

[226] Chen S (2023). Macrophages in immunoregulation and therapeutics. Signal Transduct. Target Ther..

[227] Xiang X, Wang J, Lu D, Xu X (2021). Targeting tumor-associated macrophages to synergize tumor immunotherapy. Signal Transduct. Target Ther..

[228] Byles V (2013). The TSC-mTOR pathway regulates macrophage polarization. Nat. Commun..

[229] Linke M (2017). mTORC1 and mTORC2 as regulators of cell metabolism in immunity. FEBS Lett..

[230] Park SR, Yoo YJ, Ban YH, Yoon YJ (2010). Biosynthesis of rapamycin and its regulation: past achievements and recent progress. J. Antibiot..

[231] Hobby G, Clark R, Woywodt A (2022). A treasure from a barren island: the discovery of rapamycin. Clin. Kidney J..

[232] Ballou LM, Lin RZ (2008). Rapamycin and mTOR kinase inhibitors. J. Chem. Biol..

[233] Schreiber KH (2015). Rapamycin-mediated mTORC2 inhibition is determined by the relative expression of FK506-binding proteins. Aging Cell.

[234] Veroux M (2013). Exploring new frontiers: sirolimus as a pharmacokinetic modulator in advanced cancer patients. Expert Rev. Anticancer Ther..

[235] Bhat M, Sonenberg N, Gores GJ (2013). The mTOR pathway in hepatic malignancies. Hepatology.

[236] Sehgal SN (2003). Sirolimus: its discovery, biological properties, and mechanism of action. Transpl. Proc..

[237] Lamming DW (2016). Inhibition of the mechanistic target of rapamycin (mTOR)-rapamycin and beyond. Cold Spring Harb.Perspect. Med..

[238] Zhou Y (2016). Sirolimus induces apoptosis and reverses multidrug resistance in human osteosarcoma cells in vitro via increasing microRNA-34b expression. Acta Pharm. Sin..

[239] Cohen EE (2012). Phase I studies of sirolimus alone or in combination with pharmacokinetic modulators in advanced cancer patients. Clin. Cancer Res..

[240] Aspeslet LJ, Yatscoff RW (2000). Requirements for therapeutic drug monitoring of sirolimus, an immunosuppressive agent used in renal transplantation. Clin. Ther..

[241] McCormack FX (2011). Efficacy and safety of sirolimus in lymphangioleiomyomatosis. N. Engl. J. Med..

[242] Bissler JJ (2008). Sirolimus for angiomyolipoma in tuberous sclerosis complex or lymphangioleiomyomatosis. N. Engl. J. Med..

[243] Wagner AJ (2021). nab-Sirolimus for patients with malignant perivascular epithelioid cell tumors. J. Clin. Oncol..

[244] Gordon EM (2023). A phase I/II investigation of safety and efficacy of nivolumab and nab-sirolimus in patients with a variety of tumors with genetic mutations in the mTOR pathway. Anticancer Res..

[245] Cirstea D (2010). Dual inhibition of akt/mammalian target of rapamycin pathway by nanoparticle albumin-bound-rapamycin and perifosine induces antitumor activity in multiple myeloma. Mol. Cancer Ther..

[246] Guduru SKR, Arya P (2018). Synthesis and biological evaluation of rapamycin-derived, next generation small molecules. Medchemcomm..

[247] Zanardi E (2015). Clinical experience with temsirolimus in the treatment of advanced renal cell carcinoma. Ther. Adv. Urol..

[248] Kwitkowski VE (2010). FDA approval summary: temsirolimus as treatment for advanced renal cell carcinoma. Oncologist.

[249] Hudes G (2007). Temsirolimus, interferon alfa, or both for advanced renal-cell carcinoma. N. Engl. J. Med..

[250] Atkins MB (2004). Randomized phase II study of multiple dose levels of CCI-779, a novel mammalian target of rapamycin kinase inhibitor, in patients with advanced refractory renal cell carcinoma. J. Clin. Oncol..

[251] Ali ES (2022). Recent advances and limitations of mTOR inhibitors in the treatment of cancer. Cancer Cell Int..

[252] Emons G (2016). Temsirolimus in women with platinum-refractory/resistant ovarian cancer or advanced/recurrent endometrial carcinoma. A phase II study of the AGO-study group (AGO-GYN8). Gynecol. Oncol..

[253] Tinker AV (2013). Phase II study of temsirolimus (CCI-779) in women with recurrent, unresectable, locally advanced or metastatic carcinoma of the cervix. A trial of the NCIC Clinical Trials Group (NCIC CTG IND 199). Gynecol. Oncol..

[254] Rheingold SR (2017). A phase 1 trial of temsirolimus and intensive re-induction chemotherapy for 2nd or greater relapse of acute lymphoblastic leukaemia: a Children’s Oncology Group study (ADVL1114). Br. J. Haematol..

[255] Tasian SK (2022). Temsirolimus combined with cyclophosphamide and etoposide for pediatric patients with relapsed/refractory acute lymphoblastic leukemia: a therapeutic advances in childhood leukemia consortium trial (TACL 2014-001). Haematologica.

[256] Barata PC (2019). Phase I/II study evaluating the safety and clinical efficacy of temsirolimus and bevacizumab in patients with chemotherapy refractory metastatic castration-resistant prostate cancer. Invest. N. Drugs.

[257] Schuler W (1997). SDZ RAD, a new rapamycin derivative: pharmacological properties in vitro and in vivo. Transplantation.

[258] Lee L, Ito T, Jensen RT (2018). Everolimus in the treatment of neuroendocrine tumors: efficacy, side-effects, resistance, and factors affecting its place in the treatment sequence. Expert Opin. Pharmacother..

[259] O’Shaughnessy J, Thaddeus Beck J, Royce M (2018). Everolimus-based combination therapies for HR+, HER2- metastatic breast cancer. Cancer Treat. Rev..

[260] Hasskarl J (2018). Everolimus. Small Mol. Oncol..

[261] Kanesvaran R (2015). A single-arm phase 1b study of everolimus and sunitinib in patients with advanced renal cell carcinoma. Clin. Genitourin. Cancer.

[262] Molina AM (2012). Phase 1 trial of everolimus plus sunitinib in patients with metastatic renal cell carcinoma. Cancer.

[263] Powles T (2016). A randomised phase 2 study of AZD2014 versus everolimus in patients with vegf-refractory metastatic clear cell renal cancer. Eur. Urol..

[264] Schneider TC (2017). Everolimus in patients with advanced follicular-derived thyroid cancer: results of a phase II clinical trial. J. Clin. Endocrinol. Metab..

[265] Casadevall D (2022). mTOR inhibition and T-DM1 in HER2-positive breast cancer. Mol. Cancer Res..

[266] Colon-Otero G (2017). Phase 2 trial of everolimus and letrozole in relapsed estrogen receptor-positive high-grade ovarian cancers. Gynecol. Oncol..

[267] Barnes JA (2013). Everolimus in combination with rituximab induces complete responses in heavily pretreated diffuse large B-cell lymphoma. Haematologica.

[268] Krueger DA (2010). Everolimus for subependymal giant-cell astrocytomas in tuberous sclerosis. N. Engl. J. Med..

[269] Yao JC (2011). Everolimus for advanced pancreatic neuroendocrine tumors. N. Engl. J. Med..

[270] Yao JC (2016). Everolimus for the treatment of advanced, non-functional neuroendocrine tumours of the lung or gastrointestinal tract (RADIANT-4): a randomised, placebo-controlled, phase 3 study. Lancet.

[271] Chawla SP (2012). Phase II study of the mammalian target of rapamycin inhibitor ridaforolimus in patients with advanced bone and soft tissue sarcomas. J. Clin. Oncol..

[272] Demetri GD (2013). Results of an international randomized phase III trial of the mammalian target of rapamycin inhibitor ridaforolimus versus placebo to control metastatic sarcomas in patients after benefit from prior chemotherapy. J. Clin. Oncol..

[273] Oza AM (2015). Randomized phase II trial of ridaforolimus in advanced endometrial carcinoma. J. Clin. Oncol..

[274] Seiler M (2015). Oral ridaforolimus plus trastuzumab for patients with HER2+ trastuzumab-refractory metastatic breast cancer. Clin. Breast Cancer.

[275] Rizzieri DA (2008). A phase 2 clinical trial of deforolimus (AP23573, MK-8669), a novel mammalian target of rapamycin inhibitor, in patients with relapsed or refractory hematologic malignancies. Clin. Cancer Res..

[276] Frappaz D (2016). Phase 1 study of dalotuzumab monotherapy and ridaforolimus-dalotuzumab combination therapy in paediatric patients with advanced solid tumours. Eur. J. Cancer.

[277] Pearson AD (2016). A phase 1 study of oral ridaforolimus in pediatric patients with advanced solid tumors. Oncotarget.

[278] Banerjee S (2023). Efficacy and safety of weekly paclitaxel plus vistusertib vs paclitaxel alone in patients with platinum-resistant ovarian high-grade serous carcinoma: the OCTOPUS multicenter, phase 2, randomized clinical trial. JAMA Oncol..

[279] Xu W (2023). mTOR inhibition amplifies the anti-lymphoma effect of PI3Kbeta/delta blockage in diffuse large B-cell lymphoma. Leukemia.

[280] Li Q (2013). The dual mTORC1 and mTORC2 inhibitor AZD8055 inhibits head and neck squamous cell carcinoma cell growth in vivo and in vitro. Biochem Biophys. Res. Commun..

[281] Hu W (2023). Anti-tumor effect of AZD8055 against bladder cancer and bladder cancer-associated macrophages. Heliyon.

[282] Willems L (2012). The dual mTORC1 and mTORC2 inhibitor AZD8055 has anti-tumor activity in acute myeloid leukemia. Leukemia.

[283] Naing A (2012). Safety, tolerability, pharmacokinetics and pharmacodynamics of AZD8055 in advanced solid tumours and lymphoma. Br. J. Cancer.

[284] Chen Y (2018). AZD8055 exerts antitumor effects on colon cancer cells by inhibiting mTOR and cell-cycle progression. Anticancer Res..

[285] Carracedo A (2008). Inhibition of mTORC1 leads to MAPK pathway activation through a PI3K-dependent feedback loop in human cancer. J. Clin. Investig..

[286] Ilagan E, Manning BD (2016). Emerging role of mTOR in the response to cancer therapeutics. Trends Cancer.

[287] Zeng Z (2007). Rapamycin derivatives reduce mTORC2 signaling and inhibit AKT activation in AML. Blood.

[288] Zheng Y, Jiang Y (2015). mTOR inhibitors at a glance. Mol. Cell Pharm..

[289] Wu X (2022). Recent advances in dual PI3K/mTOR inhibitors for tumour treatment. Front. Pharm..

[290] Kanojia D (2017). Kinase profiling of liposarcomas using RNAi and drug screening assays identified druggable targets. J. Hematol. Oncol..

[291] Hassan B (2014). Catalytic mTOR inhibitors can overcome intrinsic and acquired resistance to allosteric mTOR inhibitors. Oncotarget.

[292] LoRusso PM (2016). Inhibition of the PI3K/AKT/mTOR pathway in solid tumors. J. Clin. Oncol..

[293] Fingar DC (2004). mTOR controls cell cycle progression through its cell growth effectors S6K1 and 4E-BP1/eukaryotic translation initiation factor 4E. Mol. Cell Biol..

[294] Rodrik-Outmezguine VS (2011). mTOR kinase inhibition causes feedback-dependent biphasic regulation of AKT signaling. Cancer Discov..

[295] Yang J (2019). Targeting PI3K in cancer: mechanisms and advances in clinical trials. Mol. Cancer.

[296] Fan Q (2017). A kinase inhibitor targeted to mTORC1 drives regression in glioblastoma. Cancer Cell.

[297] Slotkin EK (2015). MLN0128, an ATP-competitive mTOR kinase inhibitor with potent in vitro and in vivo antitumor activity, as potential therapy for bone and soft-tissue sarcoma. Mol. Cancer Ther..

[298] Gokmen-Polar Y (2012). Investigational drug MLN0128, a novel TORC1/2 inhibitor, demonstrates potent oral antitumor activity in human breast cancer xenograft models. Breast Cancer Res Treat..

[299] Hernandez-Prat A (2019). Novel oral mTORC1/2 inhibitor TAK-228 has synergistic antitumor effects when combined with paclitaxel or pi3kalpha inhibitor TAK-117 in preclinical bladder cancer models. Mol. Cancer Res..

[300] Moore KN (2018). Phase I study of the investigational oral mTORC1/2 inhibitor sapanisertib (TAK-228): tolerability and food effects of a milled formulation in patients with advanced solid tumours. ESMO Open.

[301] Voss MH (2020). Phase 1 study of mTORC1/2 inhibitor sapanisertib (TAK-228) in advanced solid tumours, with an expansion phase in renal, endometrial or bladder cancer. Br. J. Cancer.

[302] Graham L (2018). A phase II study of the dual mTOR inhibitor MLN0128 in patients with metastatic castration resistant prostate cancer. Invest. N. Drugs.

[303] Riess JW (2021). Phase 1 trial of MLN0128 (Sapanisertib) and CB-839 HCl (Telaglenastat) in patients with advanced NSCLC (NCI 10327): rationale and study design. Clin. Lung Cancer.

[304] Zhang S (2017). Pan-mTOR inhibitor MLN0128 is effective against intrahepatic cholangiocarcinoma in mice. J. Hepatol..

[305] Caro-Vegas, C. et al. Targeting mTOR with MLN0128 overcomes rapamycin and chemoresistant primary effusion lymphoma. *mBio* **10**, 10–1128 (2019). 10.1128/mBio.02871-18 PMC6381283 30782662

[306] Shang R (2021). Cabozantinib-based combination therapy for the treatment of hepatocellular carcinoma. Gut.

[307] Badawi M (2018). CD44 positive and sorafenib insensitive hepatocellular carcinomas respond to the ATP-competitive mTOR inhibitor INK128. Oncotarget.

[308] Fricke SL (2019). MTORC1/2 inhibition as a therapeutic strategy for PIK3CA mutant cancers. Mol. Cancer Ther..

[309] Chamberlain CE (2018). A patient-derived xenograft model of pancreatic neuroendocrine tumors identifies sapanisertib as a possible new treatment for everolimus-resistant tumors. Mol. Cancer Ther..

[310] Fiorentino F, Krell J, de la Rosa CN, Webber L (2022). DICE: dual mTorc inhibition in advanced/recurrent epithelial ovarian cancer resistant to standard treatment-a study protocol for a randomised trial investigating a novel therapy called TAK228. Trials.

[311] Wang JY, Jin X, Zhang X, Li XF (2018). CC-223 inhibits human head and neck squamous cell carcinoma cell growth. Biochem. Biophys. Res Commun..

[312] Xie Z (2017). CC-223 blocks mTORC1/C2 activation and inhibits human hepatocellular carcinoma cells in vitro and in vivo. PLoS ONE.

[313] Jin Z (2017). Preclinical study of CC223 as a potential anti-ovarian cancer agent. Oncotarget.

[314] Wolin E (2019). A phase 2 study of an oral mTORC1/mTORC2 kinase inhibitor (CC-223) for non-pancreatic neuroendocrine tumors with or without carcinoid symptoms. PLoS ONE.

[315] Bhagwat SV (2011). Preclinical characterization of OSI-027, a potent and selective inhibitor of mTORC1 and mTORC2: distinct from rapamycin. Mol. Cancer Ther..

[316] Srivastava RK (2022). Combined inhibition of BET bromodomain and mTORC1/2 provides therapeutic advantage for rhabdomyosarcoma by switching cell death mechanism. Mol. Carcinog..

[317] Lou J, Lv JX, Zhang YP, Liu ZJ (2022). OSI-027 inhibits the tumorigenesis of colon cancer through mediation of c-Myc/FOXO3a/PUMA axis. Cell Biol. Int..

[318] Xu E (2021). OSI-027 alleviates oxaliplatin chemoresistance in gastric cancer cells by suppressing P-gp induction. Curr. Mol. Med..

[319] Zhi X (2015). OSI-027 inhibits pancreatic ductal adenocarcinoma cell proliferation and enhances the therapeutic effect of gemcitabine both in vitro and in vivo. Oncotarget.

[320] Chen B (2015). The antipancreatic cancer activity of OSI-027, a potent and selective inhibitor of mTORC1 and mTORC2. DNA Cell Biol..

[321] He Y (2021). Targeting PI3K/Akt signal transduction for cancer therapy. Signal Transduct. Target Ther..

[322] Chiarini F, Evangelisti C, McCubrey JA, Martelli AM (2015). Current treatment strategies for inhibiting mTOR in cancer. Trends Pharm. Sci..

[323] Bhende PM (2010). The dual PI3K/mTOR inhibitor, NVP-BEZ235, is efficacious against follicular lymphoma. Leukemia.

[324] Massard C (2017). Phase Ib dose-finding study of abiraterone acetate plus buparlisib (BKM120) or dactolisib (BEZ235) in patients with castration-resistant prostate cancer. Eur. J. Cancer.

[325] Yu Z (2015). NVP-BEZ235, a novel dual PI3K-mTOR inhibitor displays anti-glioma activity and reduces chemoresistance to temozolomide in human glioma cells. Cancer Lett..

[326] Lu X (2017). Effective combinatorial immunotherapy for castration-resistant prostate cancer. Nature.

[327] Calero R, Morchon E, Martinez-Argudo I, Serrano R (2017). Synergistic anti-tumor effect of 17AAG with the PI3K/mTOR inhibitor NVP-BEZ235 on human melanoma. Cancer Lett..

[328] Brown JR (2018). Voxtalisib (XL765) in patients with relapsed or refractory non-Hodgkin lymphoma or chronic lymphocytic leukaemia: an open-label, phase 2 trial. Lancet Haematol..

[329] Tarantelli C (2018). PQR309 is a novel dual PI3K/mTOR inhibitor with preclinical antitumor activity in lymphomas as a single agent and in combination therapy. Clin. Cancer Res..

[330] Yuan J (2011). PF-04691502, a potent and selective oral inhibitor of PI3K and mTOR kinases with antitumor activity. Mol. Cancer Ther..

[331] Jackson ER (2023). ONC201 in combination with paxalisib for the treatment of H3K27-altered diffuse midline glioma. Cancer Res..

[332] Szklener, K. et al. New directions in the therapy of glioblastoma. *Cancers* **14**, 5377 (2022). 10.3390/cancers14215377 PMC9655599 36358795

[333] Wen PY (2020). First-in-human phase I study to evaluate the brain-penetrant PI3K/mTOR inhibitor GDC-0084 in patients with progressive or recurrent high-grade glioma. Clin. Cancer Res..

[334] Ellingson BM (2020). Multiparametric MR-PET imaging predicts pharmacokinetics and clinical response to GDC-0084 in patients with recurrent high-grade glioma. Clin. Cancer Res..

[335] Jang DK (2020). GDC-0980 (apitolisib) treatment with gemcitabine and/or cisplatin synergistically reduces cholangiocarcinoma cell growth by suppressing the PI3K/Akt/mTOR pathway. Biochem. Biophys. Res. Commun..

[336] Omeljaniuk, W. J. et al. Novel dual PI3K/mTOR inhibitor, apitolisib (GDC-0980), inhibits growth and induces apoptosis in human glioblastoma cells. *Int. J. Mol. Sci*. **22**, 11511 (2021). 10.3390/ijms222111511 PMC8583746 34768941

[337] Dolly SO (2016). Phase I study of apitolisib (GDC-0980), dual phosphatidylinositol-3-kinase and mammalian target of rapamycin kinase inhibitor, in patients with advanced solid tumors. Clin. Cancer Res..

[338] Mehnert JM (2018). A phase I dose-escalation study of the safety and pharmacokinetics of a tablet formulation of voxtalisib, a phosphoinositide 3-kinase inhibitor, in patients with solid tumors. Invest. N. Drugs.

[339] Tutak I, Ozdil B, Uysal A (2022). Voxtalisib and low intensity pulsed ultrasound combinatorial effect on glioblastoma multiforme cancer stem cells via PI3K/AKT/mTOR. Pathol. Res. Pr..

[340] Arend RC (2020). EMR 20006-012: a phase II randomized double-blind placebo controlled trial comparing the combination of pimasertib (MEK inhibitor) with SAR245409 (PI3K inhibitor) to pimasertib alone in patients with previously treated unresectable borderline or low grade ovarian cancer. Gynecol. Oncol..

[341] Wen PY (2015). Phase I dose-escalation study of the PI3K/mTOR inhibitor voxtalisib (SAR245409, XL765) plus temozolomide with or without radiotherapy in patients with high-grade glioma. Neuro Oncol..

[342] Beaufils F (2017). 5-(4,6-Dimorpholino-1,3,5-triazin-2-yl)-4-(trifluoromethyl)pyridin-2-amine (PQR309), a potent, brain-penetrant, orally bioavailable, pan-class I PI3K/mTOR inhibitor as clinical candidate in oncology. J. Med. Chem..

[343] Hsin, I. L. et al. Suppression of PI3K/Akt/mTOR/c-Myc/mtp53 positive feedback loop induces cell cycle arrest by dual PI3K/mTOR inhibitor PQR309 in endometrial cancer cell lines. *Cells* **10**, 2916 (2021). 10.3390/cells10112916 PMC8616154 34831139

[344] Smith MC (2016). Characterization of LY3023414, a novel PI3K/mTOR dual inhibitor eliciting transient target modulation to impede tumor growth. Mol. Cancer Ther..

[345] Sakamoto Y (2018). PI3K-mTOR pathway identified as a potential therapeutic target in biliary tract cancer using a newly established patient-derived cell panel assay. Jpn J. Clin. Oncol..

[346] Du J (2021). The PI3K/mTOR inhibitor ompalisib suppresses nonhomologous end joining and sensitizes cancer cells to radio- and chemotherapy. Mol. Cancer Res..

[347] Lukey, P. T. et al. A randomised, placebo-controlled study of omipalisib (PI3K/mTOR) in idiopathic pulmonary fibrosis. *Eur. Respir. J*. **53**, 1801992 (2019). 10.1183/13993003.01992-2018 30765508

[348] Leiker AJ (2015). Radiation enhancement of head and neck squamous cell carcinoma by the dual PI3K/mTOR inhibitor PF-05212384. Clin. Cancer Res..

[349] Liu T (2015). Dual PI3K/mTOR inhibitors, GSK2126458 and PKI-587, suppress tumor progression and increase radiosensitivity in nasopharyngeal carcinoma. Mol. Cancer Ther..

[350] Colombo I (2021). Phase I dose-escalation study of the dual PI3K-mTORC1/2 inhibitor gedatolisib in combination with paclitaxel and carboplatin in patients with advanced solid tumors. Clin. Cancer Res..

[351] Curigliano G (2023). A Phase 1B open-label study of gedatolisib (PF-05212384) in combination with other anti-tumour agents for patients with advanced solid tumours and triple-negative breast cancer. Br. J. Cancer.

[352] Del Campo JM (2016). A randomized phase II non-comparative study of PF-04691502 and gedatolisib (PF-05212384) in patients with recurrent endometrial cancer. Gynecol. Oncol..

[353] Wainberg ZA (2017). A multi-arm phase I study of the PI3K/mTOR inhibitors PF-04691502 and gedatolisib (PF-05212384) plus irinotecan or the MEK inhibitor PD-0325901 in advanced cancer. Target Oncol..

[354] Huang, Y. et al. Novel nanococktail of a dual PI3K/mTOR inhibitor and cabazitaxel for castration-resistant prostate cancer. *Adv. Ther.* **3**, 2000075 (2020). 10.1002/adtp.202000075 PMC7567330 33072858

[355] Shapiro GI (2015). First-in-human study of PF-05212384 (PKI-587), a small-molecule, intravenous, dual inhibitor of PI3K and mTOR in patients with advanced cancer. Clin. Cancer Res..

[356] Liu C (2021). ABCB1 and ABCG2 restricts the efficacy of gedatolisib (PF-05212384), a PI3K inhibitor in colorectal cancer cells. Cancer Cell Int..

[357] Mallon R (2011). Antitumor efficacy of PKI-587, a highly potent dual PI3K/mTOR kinase inhibitor. Clin. Cancer Res..

[358] Wang Y (2019). Metformin improves mitochondrial respiratory activity through activation of AMPK. Cell Rep..

[359] Wang Y (2018). Metformin induces autophagy and G0/G1 phase cell cycle arrest in myeloma by targeting the AMPK/mTORC1 and mTORC2 pathways. J. Exp. Clin. Cancer Res..

[360] Dowling RJ (2007). Metformin inhibits mammalian target of rapamycin-dependent translation initiation in breast cancer cells. Cancer Res..

